# Quantifying the functional disparity in pigment spot-background egg colour ICP-OES-based eggshell ionome at two extremes of avian embryonic development

**DOI:** 10.1038/s41598-020-79040-4

**Published:** 2020-12-17

**Authors:** Grzegorz Orłowski, Przemysław Niedzielski, Dorota Merta, Przemysław Pokorny, Jędrzej Proch

**Affiliations:** 1 Institute for Agricultural and Forest Environment, Polish Academy of Sciences, Bukowska 19, 60-809 Poznań, Poland; 2 Department of Analytical Chemistry, Adam Mickiewicz University, Uniwersytetu Poznańskiego 8, 61-614 Poznań, Poland; 3 Department of Ecology and Environmental Protection, Pedagogical University of Kraków, Podchorążych 2, 30-084 Kraków, Poland; 4 Department of Limnology and Fishery, Institute of Animal Breeding, Wrocław University of Environmental and Life Sciences, Chełmońskiego 38C, 51-630 Wrocław, Poland

**Keywords:** Embryogenesis, Ecophysiology

## Abstract

It is known that a developing avian embryo resorbs micronutrients (calcium and other chemical elements) from the inner layer of the eggshell, inducing thinning and overall changes in the shell’s chemical composition. However, an aspect yet to be explored relates to the local changes in the multi-elemental composition (*ionome*) of the pigment spot and adjacent background colour regions of eggshells resulting from avian embryogenesis (with respect to two extremes of embryonic growth: the maternal level at the moment of egg laying, and after the completion of embryonic growth). To address this problem, we used inductively-coupled plasma optical emission spectrometry (ICP-OES) to establish the elemental profiles of microsamples from the cryptic eggs of Capercaillie *Tetrao urogallus* and Black Grouse *Tetrao tetrix*, representing the background colour and pigment spot regions of the shell. We then related these to the developmental stage of the eggs (non-embryonated eggs vs. post-hatched eggshells) and their origin (wild vs. captive hens). Our results show an apparent local disparity between the pigment spot and background colour regions in the distribution of chemical elements: most elements tended to be at higher levels in the speckled regions of the shell, these differences becoming less pronounced in post-hatched eggshells. The trends of changes following embryonic eggshell etching between the pigment spot and background colour shell regions were conflicting and varied between the two species. We hypothesized that one potential working explanation for these interspecific differences could be based on the variable composition of elements (mostly of Ca and Mg), which are the result of the varying thickness of the individual shell layers, especially as the relative difference in shell thickness in the pigment spots and background colour regions was less in Black Grouse eggs. Overall, this investigation strongly suggests that egg maculation plays a functional role in the physiological deactivation of trace elements by incorporating them into the less calcified external shell layer but without participating in micronutrient resorption. Our major critical conclusion is that all research involving the chemical analysis of eggshells requires standardized eggshell sampling procedures in order to unify their colouration and embryonic status.

## Introduction

The avian eggshell functions as a protective housing for the developing chick embryo against mechanical or environmental impact and bacterial invasion. It also allows breeding females to produce more than their own body weight of eggs without losing the capacity for flight, controls the exchange of water and gases through pores, and is the main calcium reservoir for skeletal formation during embryogenesis (reviewed in^1–3^). The primary function of eggshells, however, is to supply Ca and other micronutrients, including minor (trace) elements, to developing avian embryos. This results in the functional and structural transformation of the calcified shell and egg interior^4–6^. Hence, knowledge of the functional changes in the chemical composition and quality (above all, the thickness) of eggshells induced by embryogenesis and the resultant etching (thinning) of the innermost shell layer by developing chick embryos^7^, as well as studies of the individual within-egg variation in these features, are essential for a proper interpretation of the results of a variety of ecological, ecophysiological and ecotoxicological investigations of birds’ eggs^8–10^.

Just as importantly, laboratory and field studies have indicated that elevated levels of individual trace elements/metals, e.g. Hg, Pb, Co, Cu, Ni and Al, can variously influence eggshell quality, mostly by impairing eggshell formation and causing eggshell thinning. Such high amounts can also affect eggshell mineralization in three fundamental ways (after^1^): they reduce the availability of calcium in the diet (e.g. Ni, Co, Cu, Al), disrupt Ca metabolism (as Pb competes with Ca and interferes in Ca-mediated processes), and can interfere (e.g. Cu, Zn, Pb, and Fe) with the mineralization process itself by affecting the precipitation rate, mineralogy, size and morphology of the crystals that make up the eggshell. The most evident manifestation of trace metal contamination is the reduction of eggshell thickness (up to 17%) at such contaminated sites^1,11,12^. These metals are also transferred at different intensities from the female body to the egg contents and shells^13^. Therefore, treating each micronutrient element independently is justified in the context of physiology, its potential functions and its importance for avian reproduction. That is why in studies of the metallobiology of avian eggshells (and almost all other ecotoxicological studies), each individual metal has traditionally been treated separately^1,13–16^.

There is a growing body of evidence to show that the speckling (maculation, pigment spotting) of avian eggshells plays a functional role in embryonic growth, in particular, as regards temperature regulation, limitation of the UV-B radiation reaching the egg content (reviewed in^17^), and shell reinforcement^13^. The distribution of dark (reddish or brown) pigments within the shell’s thickness, however, is taxonomically very varied^19^. They can be (1) distributed within the outermost layer of the shell, thereby increasing its overall thickness (as in Galliformes^20^, this study); (2) embedded within the shell, demarcating a thinner shell region (as in Passerines^18^); and (3) internalized within the middle part of its thickness (Falconiformes^21^). A central question in the developmental biology of birds has yet to be addressed, namely, the role of maculation in eggs at the maternal level, i.e. at the moment of egg laying, and the subsequent resorption of shell material (mammillary body) or micronutrients from this particular region of the shell (in many species distinct in colour and thickness from the adjacent background colour region; see “Results”) during embryogenesis. It is known that female birds can distribute micronutrients and trace elements into pigment spots and unpigmented regions of an eggshell to varying extents^16^. This may be exemplified by the Japanese Quail *Coturnix coturnix japonica*, in whose eggs the levels of both major (Ca and Mg) and minor/trace elements (Cu and Cd) were found to be significantly higher in the speckled than in the adjacent unpigmented eggshell areas^16^. In contrast, Pb peaked in the plain areas of eggshells compared with the speckled ones, and the concentrations of Mn, Fe and Co measured in the speckled and unpigmented eggshell regions were variable and dependent on the egg colouration^16^. However, it is not known whether the variation in elemental composition between adjacent pigment spot and background colour regions of shells, determined in eggs representing the same developmental stage, is physiologically programmed or varies between related species that lay eggs with a similarly cryptic colouration. It should be borne in mind that even a speckled eggshell region shows a higher affinity for certain chemical elements, mostly through their ability to bind to pigment (protoporphyrin) molecules. The pool of metals present in speckled shell regions—located in the outermost shell layers and not resorbed by developing embryos^7^—does not contribute to embryonic growth by providing micronutrient/chemical elements. On the other hand, there is ample evidence for a direct link between the darker pigmentation of eggs as a result of increased pollution^21,22^ and/or the poor physiological condition of females^23,24^. Finally, it is worth stressing that the incorporation of certain chemical elements into a speckled shell region may be a physiological mechanism enabling female birds to excrete excesses of some trace elements^25^. Originally, however, this idea did not distinguish within-egg differences in the accumulation of elements relating to the presence of pigment spots or changes in the elemental composition of eggshells during embryogenesis.

Importantly, the expression of the within-egg functional variation in specific egg features is that avian eggshells are not chemically homogeneous throughout their thickness, especially as the innermost shell layer (subject to embryonic etching) is richer in Ca, whereas the outer, non-eroding, shell layer contains more Mg^26,27^. During embryogenesis, therefore, the levels of individual elements in the entire eggshell can increase (Ca, Mg and Cr) or decrease (e.g. Cu, Mn, Fe, Co, Cd and Zn), as demonstrated recently for the one of the species examined in this study^28^. Furthermore, there are clear within-egg differences in shell thickness over the longitudinal section of an egg, the shell commonly being thinner at the blunt pole than at the equator and often the sharp pole^3^. Such regional within-egg variation in shell thickness, combined with Ca/Mg variation, is most likely responsible for the chemical composition of a shell, i.e. the concentrations of certain elements, being heterogeneous over its longitudinal section^29^.

Although the scientific literature is replete with investigations of the elemental composition of avian eggshells, most papers are limited to just a few basic chemical elements (apart from those listed above, other trace elements such as Mn, Na, Zn, As, Hg or Al). This is because one traditional method of chemical analysis—atomic absorption spectroscopy (AAS)—is usually applied. This enables just one element to be measured in the analyte; in combination with the small mass of the sample, often an isolated shell fragment with pigment spotting, this reduces the possibility of measuring a small group of elements. The analytical material in such studies frequently consists of differently-coloured eggshell fragments of unknown developmental status^1,25,30^. Importantly, however, there have been great advances in modern, high-throughput methods of analytical chemistry, in particular mass spectrometry-based techniques for quantitatively identifying the entire set of macromolecules and/or elements in a micro-sample of biological material, such as the proteins in birds’ eggs or eggshells—the *proteome*^31,32^—or the total elemental composition of plant tissues—the *ionome* (sensu^33^). Thus, the application of novel analytical methods to investigate the relationship between the colouration and multi-elemental composition of avian eggshells (hereafter, as in the latter paper, referred to as the *eggshell ionome*) is entirely justified. This tool is applicable to a variety of ecological, ecotoxicological or even evolutionary considerations, especially as there are no data at all on the occurrence in avian eggshells of certain minor chemical elements, such as rare earth elements (REEs). REEs can function as Ca (and to a lesser degree also Mg) analogues in biological systems^34^, become concentrated in reproductive organs/tissues, and even at relatively low levels can cause embryo death^35,36^. In view of the rising levels of REEs (which also have other serious adverse physiological effects on living organisms) in biological samples related to rapid industrialization and intensified agricultural productivity^37,38^, such cryptic inorganic pollutants need to be taken into consideration in both broad environmental investigations and determinations of unequivocal bioindicator media. But this can only be done on the basis of knowledge acquired from narrow, species-specific investigations.

**Figure 1:**
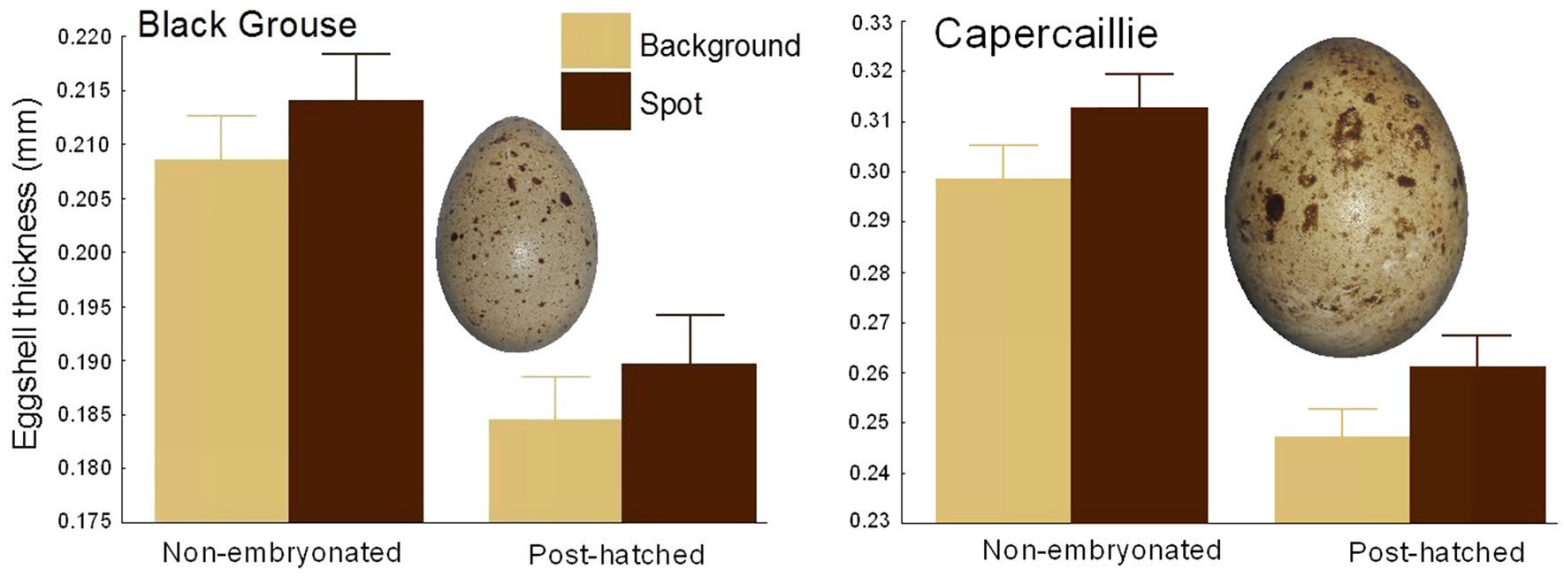
Overall eggshell thickness (average ± 95% CI) measured over the entire longitudinal section of the egg (from the sharp pole to the blunt one): both non-embryonated and post-hatched shells of the cryptic eggs of Black Grouse *Tetrao tetrix* and Capercaillie *Tetrao urogallus* (depicted as miniatures) are always significantly thinner in the background colour shell region than in the adjacent pigmented spot. *Note*: The shells of all the Black Grouse and Capercaillie eggs were respectively − 2.67% and − 5.34% thinner in the background colour than the pigment spot region. In each case, the *t*-test for paired comparisons showed highly significant differences with the exact *P* value equal to 0; see Table S1 for the statistical treatment.

Based on the above framework, we established and compared the ICP-OES-based (inductively-coupled plasma optical emission spectrometry) ionomic profiles of (micro)samples of two adjacent regions of a shell varying in colour: the background colour and the pigment spot regions. For this, we used eggs from two related species of precocial, ground-nesting birds with similar patterns of colouration (in terms of maculation and background colour; see Fig. 1). Our analysis addressed two major research goals. Firstly, we assessed the differences in elemental composition between the background colour and pigment spot regions of the eggshells at the two extremes of avian embryonic development, i.e.in non-embryonated shells (maternal effect = non-resorbed shells with the original mammillary bodies) and in post-hatched shells (with the resorbed inner layer^7^). Then we examined the potential sources of variation in the concentrations of these two local, within-egg stores of different chemical elements in the context of the developmental status and origin of these eggs. Secondly, we determined the trends of changes in elemental concentrations following embryonic eggshell etching in the maculated and background colour shell regions.

## Methods

### Study species and origin of the eggs to be analysed

In this study we used eggs from two large, ground-nesting Galliformes, Capercaillie *Tetrao urogallus* and Black Grouse *Tetrao tetrix*, collected during an extensive reintroduction programme of these species in the Bory Dolnośląskie Forest, south-western Poland^39^. Although neither species is considered to be globally threatened, many local populations in central and western Europe (like those in Poland) have become extinct, and the remaining small, isolated populations are endangered^39,40^. The catching of wild Capercaillie hens, their subsequent breeding and reproduction in captivity, and the origin/processing of their eggs/eggshells was described in detail by Orłowski et al.^28,41^. Exactly the same procedures were applied in the case of Black Grouse.

Briefly, 54 wild Capercaillie hens and 11 wild Black Grouse were caught in May 2017–2018 in northern Sweden (Västerbotten Region near Vilhelmina; 64°38′36″ N, 16°37′54″ E) and flown to Poland. On arrival, the hens were placed in roughly circular adaptation aviaries in order to acclimate them to the local conditions in Poland for the required quarantine period. During their first 2.5–3 weeks these hens laid eggs. Some eggs were laid eggs singly, in random places; these were subsequently incubated by Domestic Chickens *Gallus domestica* or in artificial incubators, but ultimately failed to hatch. The translocated hens were not stimulated to lay more eggs by having their eggs taken away from the nests; only abandoned eggs were removed^41^.

Tetraonid hens can store Ca for a relatively long time—weeks or months—prior to reproduction (through the formation and subsequent utilization of medullary bone) and then mobilize it during egg laying^42^. During the egg-laying period in Poland, the Swedish hens may have depleted the resources they had derived from their original breeding sites. Presumably, therefore, the contents of Ca and other elements/resources in the eggs laid in the acclimation aviaries were not affected by their diet in captivity. Also, contact between the wild-caught hens and males (copulation) had taken place only in Sweden. Thus, we treated the eggs laid in the incubation aviaries as if laid by wild hens^28,41^.

The eggs and post-hatched eggshells of captive-bred Capercaillie and Black Grouse were obtained from a breeding centre in Kadzidłowo (NE Poland), specializing in the rearing of these species.

Unhatched eggs were obtained after unsuccessful incubation (both natural and artificial). The normal duration of incubation is similar in both species—25–28 days^43,44^. Eggshells from hatched chicks were collected for further examination.

We examined 30 eggs/eggshells from Black Grouse—15 from wild hens and the other 15 from captive hens. The sample comprised 17 intact eggs (including one egg with a shell partly broken by the hatching chick), and 13 post-hatched eggshells. We likewise examined 35 Capercaillie eggs/eggshells—22 from wild and 13 from captive hens. This sample consisted of 8 intact eggs and 27 post-hatched eggshells. Seven of the post-hatched eggshells were sampled from wild Capercaillie living in the Augustów Primeval Forest (NE Poland; 53°50′N, 23°6′E), while the other 20 were obtained from the hens translocated from Sweden. Because two or more hens (both wild and captive-bred) were reared communally in the aviaries, precise assignment of the two categories of eggs, i.e. wild and captive, to an individual female was impossible^28,41^. This factor could not therefore be controlled for in the analysis.

### Egg processing and eggshell sampling

The unhatched eggs were opened to determine their status, and their contents poured out. After drying, the shells were stored in plastic containers at room temperature. Shell thicknesses were measured (by one co-author, PP) with the attached inner shell membrane to the nearest 10 μm using a micrometer (IP65 125–150 mm HOGETEX). The shell thickness of each egg was measured over the entire longitudinal section of the egg (from the sharp pole to the blunt one) in pairwise fashion, i.e. at a pigment spot and in the adjacent background colour region; the latter was always located within ca 1 mm from the pigment spot. The measurements for each egg were made on 3–6 pigment spots and the same number of background colour regions. However, since pigment spots were the most numerous in the equatorial shell regions, most of these measurements (and the subsequent sampling of shell fragments) were carried out in that egg region. These measurements are shown in Fig. 1.

For the chemical analysis, samples of relatively large pigment spots and the adjacent background colour regions were cut out from each eggshell using surgical instruments (tweezers, scissors, scalpel), and then pooled for each egg to obtain the appropriate sample mass. Consequently, we obtained two paired samples of eggshells from the pigmented and background colour regions of each egg, which were then chemically analysed. Worth noting is the fact that, in comparison with a previous study of ours on the elemental composition of Japanese Quail eggshells, in most of which the pigment spots were considerably larger and covered as much as 20–30% of the egg surface^16^, we obtained a larger number of (micro)samples of pigment and background colour shell regions from the Black Grouse and Capercaillie eggs for this chemical analysis, representing a more heterogeneous sampling over the longitudinal section of the egg.

### Chemical analysis

An inductively-coupled plasma optical emission spectrometer (Agilent 5110, Agilent, USA) was used to determine the elemental composition of the samples^45^. The simultaneous axial and radial views of the plasma was enabled by synchronous vertical dual view (SVDV). The standard conditions were applied: Radio Frequency (RF) power 1.2 kW, nebulizer gas flow 0.7 L min^−1^, auxiliary gas flow 1.0 L min^−1^, plasma gas flow 12.0 L min^−1^, viewing height for radial plasma observation 8 mm, detector CCD (Charge Coupled Device) temperature − 40 °C, signal accusation time 5 s for 3 replicates. A Mars 5 (CEM, Matthews, USA) microwave sample preparation system was used for sample digestion^45,46^.

Accurately weighed eggshell samples (without shell membranes) from Black Grouse (0.0152–0.3275 g; average ± SE = 0.1289 ± 0.0110 g) and from Capercaillie (0.0055–0.2990 g; average ± SE = 0.0771 ± 0.0079 g) were digested in 2 mL concentrated nitric acid in closed Teflon containers in a Mars 6 (CEM, USA) microwave sample preparation system. After digestion, the samples were diluted with water to a final volume of 6.0 mL.

The detection limits were estimated at the level of 0.01 mg kg^−1^ dry weight (ppm. d.w.) for all the elements determined (as 3-sigma criteria). The uncertainty of the overall analytical procedure (including sample preparation) was 20%. Traceability was checked using reference materials: CRM S-1—loess soil; CRM NCSDC (73349)—bush branches and leaves; CRM 2709—soil; CRM 405 and CRM 667—estuarine sediments. The recovery (80–120%) was acceptable for most of the elements determined. The recovery of the uncertified elements was defined by the standard addition method^46^. We excluded non-detects from the major quantitative analysis.

### Statistical analyses

We employed two different approaches to the data analysis in order to assess potential differences between the pigment spot and adjacent background colour regions of the eggshells (Supplementary Information: Tables S1–S7): (1) measured simultaneously as pairs (in both these regions) within an individual eggshell; and (2) collectively across all the eggshells. Extensive supplementary information on the ICP-OES-based eggshell ionomics we obtained is provided at the end of this article (see Supplementary Material).

Firstly, to achieve consistent results in the context of individual (within-egg/eggshell) variation, the data on shell thickness and ICP-OES-based eggshell ionomics were analysed as pairs, i.e. corresponding measurements for pigment spots and adjacent background colour regions within an individual eggshell. We used the *t*-test for dependent samples to test for the differences between these two regions (summarized in Tables S6,S7). The major aim of our *t*-test analysis was to assess the potential differences in ICP-OES-based ionomic data measured in the background colour and pigment spot regions between just the two main samples of eggshells (non-embryonated eggs vs. post-hatched eggshells). Importantly, our earlier paper had already documented an analogous relationship—that there were significant differences in the concentrations of eight chemical elements between the pigment spots and unpigmented shell areas—in non-embryonated maculated eggs of other gallinaceous species^16^. Formally, therefore, this earlier study^16^ should be treated as having yielded preliminary outcomes, which were specified beforehand (sensu^47^). Thus, correction for multiplicity or a multiple hypothesis test correction seem to be too conservative and should be avoided^47,48^. For each *t*-test result, we always give the actual *P* value to four decimal points (Tables S6,S7). So, as recommended, the reader can re-assess our results in the context of future research and its repeatability^48–50^. For documentation and educational purposes, Tables S6,S7 also list the results of *t*-tests for pooled groups of non-embryonated eggs and post-hatched eggshells. The major justification for this is that the developmental status of eggs was often unknown in studies of the elemental composition of avian eggs/eggshells (e.g.^1,25,30^).

**Figure 2:**
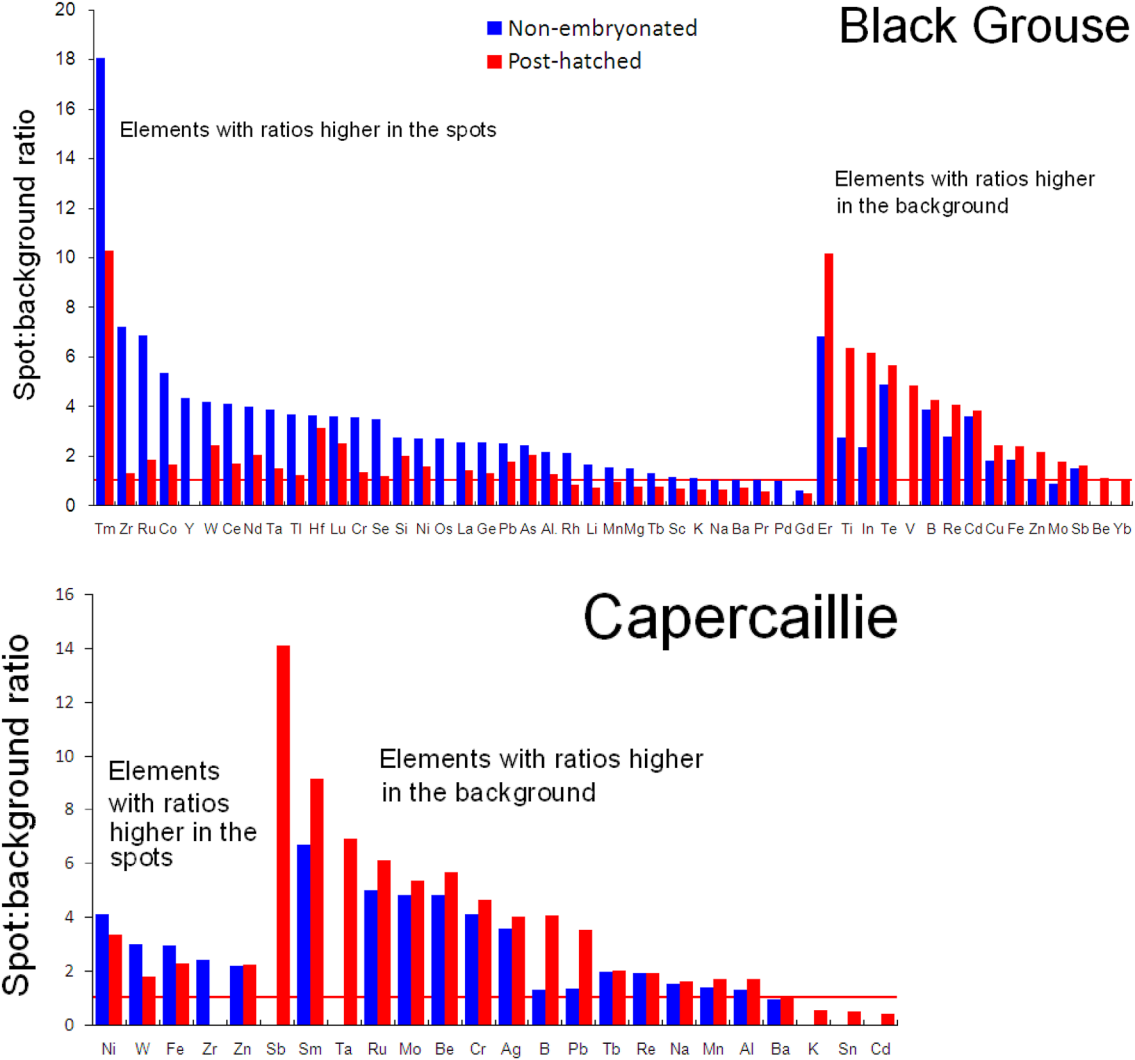
Ratios of elemental concentrations (based on pooled data for all the eggs—see Table S2 and Table S3) between the pigment spot and background colour regions of eggshells at two extremes of avian embryonic development, defined as non-embryonated eggs (maternal level) and post-hatched (resorbed) eggshells in cryptic eggs of Black Grouse *Tetrao tetrix* and Capercaillie *Tetrao urogallus*. The variation in elemental concentrations in the spot-background eggshell regions within the same eggs (for corresponding pairs of measurements) is illustrated in Fig. S1. *Note*: The red line (ratio = 1) signifies no difference in eggshell elemental concentration between the pigment spot and background colour region; the bars with ratios < 1 indicate higher elemental concentrations in the background colour region.

Secondly, owing to the large number of non-detects among the paired data, we increased the sample size by examining the overall differences in levels of individual elements between the pigment spot and adjacent background colour regions across all the available ICP-OES-based eggshell ionomics data (summarized in Tables S2–S5). For an initial visualization of the potential disparity in accumulation of all eggshell elements in the pigment spot (S) and background colour (B) regions, we used the ratios of these two (S/B) concentrations (Fig. 2).

Thereafter, the overall data were analysed (three-way or two-way full factorial ANOVAs depending on the species-specific data; see “Results”) to examine the sources of variation in eggshell elemental concentrations: the origin of the eggs (wild vs. captive hens), sample (background colour vs. pigment spot region), egg status (non-embryonated eggs vs. post-hatched eggshells) and the interaction terms between them. In ANOVA, the elemental concentrations were checked for normality (Kolmogorov–Smirnov test), and if necessary, data were log-transformed to meet the parametric assumption prior to analysis.

Further, in order to achieve our second major aim, we evaluated the trends of changes in elemental concentrations following embryonic eggshell etching between the pigment spot and background colour regions. These were expressed as the %Change in elemental concentrations between non-embryonated and post-hatched eggshells in both these shell regions based on the overall data (summarized in Tables S2 and S3).

The statistical analyses were performed using Statistica ver. 12.5^51^ and Excel software. A probability of *P* < 0.05 was considered statistically significant.

### Ethics statement

All the procedures regarding this study were conducted in compliance with Polish legislation. The experiment was approved by the Local Ethics Commission for Experiments Carried Out on Animals (permit: NR 31/2010).

## Results

Macroscopic inspection of the 17 unhatched Black Grouse eggs showed that no embryonic tissues/development was visible in 12 of them, while very early chick development (< 24 h) with incipient embryonic shield formation was discernible in another four (stages 1–3 according to^52^). The embryos of precocial species do not mobilize Ca from the eggshell during the first half of incubation^5,6^. Therefore, in order to achieve the major aims of this study, both these egg groups (not varying in eggshell thickness in either the pigment spot or background colour regions: Mann–Whitney test, *U* = 833.0 and 854.0, *P* = 0.793 and 0.936; *n* = 72/24 in each case, respectively) were pooled into one class, referred to as non-embryonated eggs. The final egg of these 17 contained a completely developed, feathered embryo with a shell partly broken at hatching; this specimen was pooled with the post-hatched eggshells.

Eight unhatched Capercaillie eggs (all from wild hens) showed no signs of embryonic development; accordingly, they were classified as non-embryonated eggs.

### Eggshell thickness and overall elemental composition of the pigment spot and background colour regions

Our measurements of non-embryonated and post-hatched eggshells of cryptic, Black Grouse and Capercaillie eggs revealed highly significant differences in the thickness of the shells between their background colour and pigment spot regions (Fig. 1; Table S1): the former were respectively 2.6% and 2.8% thinner (Black Grouse) and 4.5% and 5.3% thinner (Capercaillie) than the latter in both non-embryonated and post-hatched eggshells (Fig. 1; Table S1).

Comprehensive ICP-OES analysis of 60 eggshell samples of the background colour and pigment spot regions (each *n* = 30 samples) from 30 Black Grouse eggs yielded measurements for 55 and 54chemical elements present above the detection limit in both samples, respectively (Table 1). In contrast, the levels of 27 and 25 elements, respectively, were above the detection limit in samples from the background colour and pigment spot regions (each sample *n* = 35) from 35 Capercaillie eggs (Table 1). The frequency of detectability (= number of samples with concentrations exceeding the detection limit) of individual elements varied strongly in both species (see Supplemental Material Appendix 1).

**Table 1:** Average (± 95% CI) elemental concentrations (ppm d.w.) measured by ICP-OES in two adjacent eggshell regions with contrasting colour patterns, i.e. background colour and pigment spots, over all the eggs/eggshells of Black Grouse *Tetrao tetrix* and Capercaillie *Tetrao urogallus*; the number of samples above the detection limit and ranges are given in round and square brackets, respectively; BDL—all samples below the detection limit; n.d.—not determined; significant differences (ANOVA, *P* ≤ 0.05) in elemental concentrations measured in the background colour and pigment spot regions over all eggshells are shown in bold.

Element^†^	Black Grouse		Capercaillie	
	Background (*n* = 30)	Spot (*n* = 30)	Background (*n* = 35)	Spot (*n* = 35)
Ag	BDL	2.092 (1)	**0.111 ± 0.091** (9) [0.020–0.387]	**0.445 ± 0.327** (10) [0.014–1.542]
Al	**16.28 ± 4.311** (30) [3.794–62.76]	**25.90 ± 4.754**(30) [7.943–75.71]	**20.82 ± 3.933** (35) [9.17–75.44]	**33.75 ± 6.533** (35) [4.254–80.85]
As	**1.922 ± 1.143** (16) [0.349–8.914]	**4.231 ± 2.614** (8) [0.242–8.565]	2.148 ± 8.197 (2) [1.503–2.794]	BDL
B	**0.286 ± 0.122** (30) [0.010–1.471]	**1.162 ± 0.730** (16) [0.025–4.524]	**5.689 ± 2.347** (27) [0.119–19.875]	**23.91 ± 9.66** (27) [0.12–91.86]
Ba	357.3** ± **77.95 (30) [81.2–1003.1]	329.4** ± **82.61 (30) [50.70–1036.7]	132.5 ± 30.32 (35) [44.65–414.0]	135.5 ± 30.84 (35) [31.88–402.8]
Be	0.010 (1)	0.029** ± **0.035 (5) [0.012–0.078]	**0.073 ± 0.016** (35) [0.018–0.203]	**0.387 ± 0.098** (35) [0.102–1.412]
Bi	0.120 (1)	178.0 (1)	n.d	n.d
Cd	**0.026 ± 0.009** (11) [0.011–0.051]	**0.095 ± 0.021** (16) [0.035–0.158]	10.38 (1)	4.212 (1)
Ce	**0.671 ± 0.199** (30) [0.082–2.296]	**1.842 ± 0.509** (27) [0.148–4.716]	BDL	BDL
Co	**0.151 ± 0.044** (27) [0.010–0.490]	**0.516 ± 0.196** (29) [0.017–2.460]	BDL	BDL
Cr	**0.181 ± 0.058** (30) [0.050–0.595]	**0.364 ± 0.111** (30) [0.050–1.261]	**0.615 ± 0.159** (35) [0.182–2.496]	**2.755 ± 0.789** (35) [0.481–11.55]
Cu	1.579** ± **0.356 (30) [0.611–5.567]	3.454** ± **1.532 (30) [0.861–22.18]	BDL	BDL
Dy	BDL	BDL	0.105 ± 0.057 (9) [0.028–0.266]	BDL
Er	0.016** ± **0.019 (3) [0.010–0.025]	0.131** ± **0.094 (11) [0.023–0.477]	BDL	BDL
Fe	24.21** ± **7.169 (30) [6.956–73.66]	52.61** ± **28.68 (30) [9.905–352.4]	**22.15 ± 3.345** (35) [7.427–52.19]	**53.59 ± 10.36** (35) [11.48–166.53]
Ga	BDL	0.137** ± **(1)	BDL	BDL
Gd	0.106** ± **0.029 (29) [0.018–0.420]	0.060** ± **0.046 (7) [0.013–0.157]	BDL	BDL
Ge	0.958** ± **0.563 (18) [0.032–4.789]	1.639** ± **1.638 (9) [0.020–6.482]	BDL	BDL
Hf	**0.452 ± 0.149** (29) [0.031–2.157]	**1.517 ± 0.431** (29) [0.126–4.850]	BDL	BDL
Hg	0.017** ± **0.023 (2) [0.015–0.019]	0.044 (1)	n.d	n.d
Ho	0.015** ± **0.009 (2) [0.015–0.016]	BDL	BDL	BDL
In	**0.932 ± 0.482** (10) [0.062–1.938]	**3.092 ± 1.664** (12) [0.335–8.193]	n.d	n.d
Ir	1.140 ± 1.187 (9) [0.012–5.107]	BDL	n.d	n.d
K	1204.0 ± 276.0 (30) [737.3–3869.8]	1003.4** ± **111.4 (30) [252.1–1587.7]	**550.4 ± 92.40** (30) [54.37–1412.8]	**330.7 ± 101.4** (11) [128.7–581.1]
La	0.280 ± 0.093 (30) [0.081–1.065]	0.518** ± **0.124 (30) [0.085–1.482]	BDL	BDL
Li	0.281 ± 0.343 (28) [0.010–4.766]	0.254** ± **0.211 (15) [0.023–1.530]	BDL	BDL
Lu	**0.080 ± 0.027** (29) [0.028–0.376]	**0.237 ± 0.070** (30) [0.011–0.815]	BDL	BDL
Mg	3099.0 ± 730.0 (30) [1575.4–9568.1]	3401.3** ± **356.4 (30) [1018.9–4870.1]	n.d	n.d
Mn	10.431 ± 3.494 (30) [1.265–44.64]	13.63** ± **4.971 (30) [1.03–55.92]	6.001 ± 1.391 (35) [0.930–17.97]	9.730 ± 3.570 (35) [0.963–56.32]
Mo	0.136 ± 0.050 (10) [0.032–0.260]	0.191** ± **0.112 (7) [0.012–0.298]	**1.116 ± 0.530** (32) [0.022–8.316]	**5.840 ± 3.485** (32) [0.063–52.74]
Na	1268.3 ± 228.5 (30) [831.8–3469.7]	1072.8** ± **117.8 (30) [292.4–1847.4]	**2195.5 ± 165.3** (35) [1126.8–3265.6]	**3458.6 ± 441.0** (35) [695.9–7090.5]
Nb	0.010 (1)	BDL	n.d	n.d
Nd	1.248 ± 0.266 (30) [0.256–3.510]	3.698** ± **0.826 (30) [0.842–11.923]	BDL	BDL
Ni	0.541 ± 0.177 (26) [0.118–1.786]	1.062** ± **0.507 (27) [0.140–6.938]	**4.865 ± 1.362** (35) [0.367–21.46]	**17.53 ± 4.89** (35) [1.771–64.94]
Os	0.214 ± 0.383 (4) [0.033–0.559]	0.183** ± **0.312 (3) [0.090–0.326]	BDL	BDL
Pb	**2.237 ± 0.611** (30) [0.456–7.170]	**4.691 ± 1.175** (30) [0.055–15.906]	**2.720 ± 1.001** (15) [0.313–5.950]	**7.595 ± 4.987** (9) [0.607–18.81]
Pd	0.224 ± 0.107 (18) [0.015–0.805]	0.171 (1)	BDL	BDL
Pr	44.11 ± 10.17 (30) [31.97–136.45]	34.13** ± **3.245 (30) [9.095–43.65]	n.d	n.d
Rb	BDL	30.89** ± **34.35 (9) [2.376–144.50]	n.d	n.d
Re	**0.353 ± 0.111** (19) [0.040–0.926]	**1.222 ± 0.561** (21) [0.015–4.218]	**2.464 ± 0.819** (27) [0.186–8.741]	**4.427 ± 1.340** (24) [0.098–11.13]
Rh	0.329 ± 0.073 (30) [0.038–0.886]	0.445** ± **0.139 (28) [0.052–1.398]	BDL	BDL
Ru	**0.240 ± 0.111** (26) [0.015–1.307]	**0.853 ± 0.323** (26) [0.061–3.870]	**2.259 ± 0.549** (35) [0.415–6.328]	**12.68 ± 4.137** (35) [2.609–55.15]
Sb	18.10 ± 6.246 (19) [3.544–47.161]	28.90** ± **15.39 (18) [0.047–104.23]	12.60 (1)	177.7 (1)
Sc	0.125 ± 0.029 (30) [0.073–0.352]	0.111** ± **0.013 (30) [0.024–0.171]	BDL	BDL
Se	**1.334 ± 0.442** (27) [0.168–4.214]	**2.754 ± 1.209** (23) [0.053–9.846]	n.d	n.d
Si	**12.79 ± 3.558** (30) [2.926–48.91]	**29.01 ± 12.81** (30) [5.352–189.56]	n.d	n.d
Sm	BDL	BDL	**8.443 ± 2.577** (35) [1.020–29.36]	**67.95 ± 19.85** (35) [13.31–277.8]
Sn	n.d	n.d	22.77 ± 3.699 (21) [5.537–36.60]	11.03 (1)
Ta	0.307 ± 0.196 (25) [0.019–2.416]	0.686** ± **0.234 (17) [0.101–1.492]	22.38 (1)	155.3 (1)
Tb	0.619 ± 0.147 (30) [0.342–2.089]	0.617** ± **0.097 (30) [0.080–1.122]	**0.933 ± 0.122** (35) [0.373–2.143]	**1.847 ± 0.330** (35) [0.613–5.670]
Te	**1.206 ± 0.280** (30) [0.270–3.508]	**6.328 ± 1.954** (29) [0.827–20.50]	n.d	n.d
Th	n.d	n.d	0.826 ± 0.449 (12) [0.017–1.802]	BDL
Ti	0.113 ± 0.059 (20) [0.015–0.565]	0.543** ± **0.566 (28) [0.018–7.802]	BDL	0.104 (1)
Tl	**0.723 ± 0.196** (25) [0.032–1.609]	**1.627 ± 0.597** (22) [0.157–4.986]	n.d	n.d
Tm	**0.066 ± 0.039** (11) [0.013–0.179]	**0.960 ± 0.243** (27) [0.033–2.100]	BDL	BDL
U	0.530 ± 0.195 (13) [0.117–1.127]	BDL	BDL	BDL
V	**0.036 ± 0.020** (13) [0.012–0.137]	**0.204 ± 2.356** (2) [0.019–0.389]	BDL	BDL
W	0.374 ± 0.284 (9) [0.030–1.308]	1.048** ± **0.677 (11) [0.034–3.065]	1.338 ± 0.951 (10) [0.224–4.364]	2.694 ± 3.949 (4) [0.727–6.320]
Y	0.019 ± 0.026 (3) [0.012–0.031]	0.092** ± **0.046 (13) [0.011–0.254]	BDL	BDL
Yb	0.029 (1)	0.029** ± **0.010 (16) [0.011–0.078]	BDL	BDL
Zn	4.757 ± 1.528 (30) [1.545–20.98]	7.327** ± **4.116 (30) [0.717–60.20]	**8.079 ± 1.848** (35) [1.422–22.56]	**18.01 ± 4.467** (35) [4.530–53.33]
Zr	0.230 ± 0.363 (6) [0.036–0.932]	0.561** ± **0.225 (20) [0.057–2.112]	1.478 ± 3.363 (3) [0.395–2.996]	7.620 ± 7. 322 (6) [0.448–16.38]

The results of the paired comparison of background-spot elemental concentrations for the same eggs are listed in Tables S5 and S6.

^†^Rare earth elements (REEs) are underlined.

We had anticipated that the elemental concentrations between these two adjacent shell regions would vary considerably in both the non-embryonated and post-hatched eggshells (see Supplemental Material Appendix 1 and Tables S2–S7). Further statistical treatment of these data taking into account the two extremes of embryonic growth was therefore required if we were to achieve the major goal of our study.

### Disparity in eggshell elemental composition between adjacent spotted and background colour shell regions

Analysis of the pairs of elemental concentrations measured in pigment spots and the adjacent background colour regions from the same eggshells (*t*-test for paired comparisons) showed that the concentrations of 25 of the 45 elements measured in the shells of non-embryonated eggs (Al, As, B, Ce, Co, Cr, Cu, Fe, Hf, K, La, Lu, Mg, Mn, Nd, Ni, Pb, Rh, Ru, Se, Si, Tb, Te, Tl and Tm) and just 5 of the 43 elemental concentrations measured in post-hatched Black Grouse eggshells (Hf, Mo, Nd, Te and Tm) varied significantly between these two regions (Fig. S1, Table S6). Most elemental concentrations, including those of the rare earth elements (Table S2: Ce, La, Lu, Nd, Tb and Tm), were consistently higher in the pigment spots in both the non-embryonated and post-h atched eggshells: they represented the negative values in the %Difference shown in Fig. S1. In contrast, 12 elements (Ge, Li, Zr, Pr, K, Na, Sc, Ba, Tb, Mg, Rh and Mn) were present in higher concentrations in the pigment spots of the non-embryonated eggs (Fig. S1: negative values), simultaneously exhibiting the opposite trend (Fig. S1: positive values) found in the post-hatched eggshells.

The analogous analysis of Capercaillie eggs showed that the concentrations of 8 of the 16 elements measured in the shells of non-embryonated eggs (Be, Cr, Mo, Ni, Ru, Sm, Zn and Zr), and as many as 14 of the 16 elemental concentrations measured in post-hatched shells (Al, B, Be, Cr, Fe, K, Mn, Na, Ni, Re, Ru, Sm, Tb and Zn) varied significantly between the two adjacent shell regions (Table S7). The levels of almost all these elements were higher in the pigment spots in both the non-embryonated and post-hatched eggshells; the exception was K, the level of which was higher in the background colour region of post-hatched eggshells (Fig. S1, Table S7).

However, taking into account the large number of non-detects yielding the small number of paired measurements used in the first analysis, we further evaluated the patterns of differences in elemental concentrations common to the pigment spot and background colour shell regions based on the ICP-OES-based eggshell ionomics data listed in Tables S2 and S3. In Fig. 2, therefore, we compared the spot/background (S/B) ratio of eggshell elemental concentrations between these two regions in non-embryonated and post-hatched shells. Inspection of this figure shows that most elements tended to be at higher concentrations in the speckled regions of the shell in both species, these differences being more pronounced in the post-hatched eggshells (Fig. 2; see Supplemental Material Appendix 1). The concentrations of only two elements (Gd and Mo) measured in the shells of non-embryonated Black Grouse eggs, and of 11 elements (Rh, Li, Mn, Mg,Tb, Sc, K, Na, Ba, Pr, Gd) measured in post-hatched eggshells of the same species were higher in the background colour regions (Fig. 2). Another interesting result of this analysis is that the concentrations of 33 elements from shell samples of non-embryonated eggs, listed in decreasing order of S/B ratios, are hierarchically distributed (from Tm to Gd; without Os, which was not measured in post-hatched shells) (Fig. 2), whereas 12 elements (Er, Ti, In, Te, B, Re, Cd, Cu, Fe, Zn, Mo, Sb) display higher S/B ratios in the background colour regions (Fig. 2).

### Sources of variation in eggshell elemental concentrations as a result of the developmental status and origin of eggs

Tables S4 and S5 list the elemental concentrations measured in the background colour and pigment spot regions of the eggshells, further broken down according to the origin of the eggs (wild vs. captive hens). We also explored the sources of variation in eggshell elemental concentrations in these species-specific data by using multivariate analysis to test for the effect of the origin of the eggs/eggshells, shell region, and egg status (non-embryonated eggs vs. post-hatched eggshells) and the interactions between these three factors (Tables 2, 3). The key aspect explaining our major research goal, however, concerned the sample × status and origin × sample × status interactions, which we examined in order to ascertain which factors were responsible for the variations in elemental concentrations between the background and spotted shell regions (Tables 2, 3).

**Table 2:** *F*-values and the accompanying significance levels from three-way full factorial ANOVA performed to test the effect of the origin of eggs (from wild vs. Captive hens), sample (= eggshell region—background vs. spot), egg status (non-embryonated eggs vs. post-hatched eggshells) and the interaction terms between them on ICP-OES-based eggshell elemental profiles in Black Grouse *Tetrao tetrix*.

Element	Origin	Sample	Status	Origin × sample	Origin × status	Sample × status	Origin × sample × status
Al	2.22	15.0***	5.08*	2.00	0.38	0.18	4.01*
As	6.59*	5.37*	0.12	1.24	3.35	2.30	0.05
B	12.42**	0.02	23.26***	0.01	11.18**	0.01	0.11
Ba	0.11	0.33	20.84***	0.48	0.40	2.29	0.45
Cd	19.5***	0.44	0.00	0.23	0.02	0.16	0.74
Ce	16.9***	1.39	1.06	3.45	0.002	0.13	0.24
Co	7.07*	3.36	0.09	4.50*	0.001	1.43	0.98
Cr	14.8***	7.83**	4.95*	0.02	6.84*	4.29*	3.52
Cu	5.14*	2.61	0.496	0.820	0.152	0.018	0.003
Fe	13.81***	8.84**	7.63**	4.36*	6.51*	14.62***	10.66**
Ge	1.11	0.04	0.02	0.23	0.40	0.74	1.81
Hf	31.1***	5.84*	5.26*	3.15	8.71**	0.81	1.93
K	0.66	2.49	0.82	2.39	2.30	0.09	2.66
La	11.69**	3.64	0.27	0.04	5.31*	0.01	1.20
Lu	23.15***	2.18	3.46	0.87	7.22*	0.28	2.42
Mg	1.84	0.76	0.00	2.82	4.42*	0.44	3.18
Mn	1.64	0.18	13.60***	0.42	1.59	0.18	0.29
Mo	0.92	0.44	2.25	1.55	0.01	1.89	4.82
Na	0.63	0.38	0.23	2.38	1.91	0.06	3.91
Nd	37.15***	0.32	2.74	1.23	4.66*	0.01	1.57
Ni	7.89**	6.57*	3.15	0.55	4.92*	2.10	4.39*
Pb	23.28***	5.87*	4.62*	0.80	7.56**	0.49	5.34*
Pr	1.73	1.00	1.06	2.49	2.52	0.61	2.74
Rh	3.60	0.00	0.16	2.35	2.80	0.57	1.22
Ru	11.25**	0.00	1.93	0.87	4.29*	0.16	0.01
Sb	1.65	1.46	0.05	0.02	0.09	0.05	0.25
Sc	0.07	2.06	0.03	2.37	0.79	0.00	3.04
Se	9.60**	0.36	1.93	0.32	4.50*	0.20	3.57
Si	7.00*	3.45	0.00	0.32	0.20	0.22	0.75
Ta	4.69*	0.23	0.67	1.06	0.01	0.70	0.08
Tb	0.24	2.16	0.67	1.97	0.73	0.19	2.26
Te	43.97***	1.60	7.47**	2.63	10.94**	3.19	4.19*
Ti	1.20	0.82	0.03	0.44	0.10	0.00	0.00
Tl	9.33**	0.88	1.59	4.10*	1.89	0.00	0.04
Zn	0.94	0.79	0.87	0.43	0.40	0.26	0.12

Statistically significant effects are in bold.

The elemental concentrations corresponding to the tested effects are listed in Table S3.

*P < 0.05; **P < 0.01; ***P < 0.001.

**Table 3:** *F*-values and the accompanying significance levels of two-way ANOVAs performed to test the effect of sample (= eggshell region—background vs. spot), egg status (non-embryonated eggs vs. post-hatched eggshells) and origin of eggs (from wild vs. captive birds) on ICP-OES-based eggshell elemental profiles in Capercaillie *Tetrao urogallus*.

Element	Non-embryonated and post-hatched eggshells			Post-hatched eggshells only		
	Sample	Status	Sample × status	Sample	Origin	Sample × origin
Ag	5.01*	2.44	0.70	3.66	0.31	0.13
Al	5.81*	13.37***	1.92	12.92***	0.16	0.02
B	1.38	3.59	1.35	13.87***	0.15	0.07
Ba	0.01	1.62	0.15	0.11	18.93***	0.01
Be	49.54***	11.41**	4.24*	69.80***	18.51***	12.39***
Cr	25.60***	2.68	0.76	29.25***	16.03***	9.34**
Fe	29.26***	0.44	0.87	48.03***	13.62***	5.49*
K	–	–	–	9.03**	3.89	0.84
Mn	1.83	1.53	0.32	3.27	0.48	0.21
Mo	5.84*	0.24	0.08	4.42*	4.04	2.64
Na	23.31***	5.11*	0.07	70.45***	46.87***	8.76**
Ni	36.95***	12.98**	5.91*	32.56***	7.71**	2.87
Pb	1.80	0.02	0.70	17.45***	9.39*	9.26**
Re	7.72**	6.78*	0.61	6.31*	0.19	0.82
Ru	35.73***	13.04**	5.18*	69.39***	24.96***	19.55***
Sm	46.02***	10.72**	5.06*	58.62***	17.02***	11.91**
Tb	24.39**	5.60*	0.49	39.87***	5.51*	2.96
W	1.22	0.53	0.01	0.63	0.03	0.74
Zn	18.19***	8.43**	1.11	19.39***	26.79***	4.21*

Statistically significant effects are in bold.

Since the post-hatched eggshells were obtained from only captive birds, we ran two separate ANOVAs to test (1) the effect of sample and egg status over non-embryonated and post-hatched eggshells, and (2) the effect of sample and origin within the post-hatched eggshells only. The elemental concentrations corresponding to the tested effects are listed in Table S4.

*P < 0.05; **P < 0.01; ***P < 0.001.

The analysis shows that for Black Grouse, each of the seven tested effects had a significant influence on eggshell elemental concentrations (Table 2). The sample × status interaction significantly affected the levels of just two elements—Cr and Fe (Table 2). The origin × sample × status interaction significantly influenced the levels of five elements—Al, Fe, Ni, Pb and Te (Table 2). So, linking these significant effects with the data in Table S4indicates that the eggshells of captive birds tended to have higher levels of Al, Fe, Pb and Te than those of wild Black Grouse; the levels of these elements, along with Ni, were the highest in the pigment spots.

In the case of Capercaillie, two-way ANOVA showed that the sample × status interaction in non-embryonated and post-hatched eggshells significantly influenced the levels of four elements—Be, Ni, Ru and Sm (Table 2 and Table S5), whereas the sample × origin interaction in the post-hatched eggshells only had a significant influence on the concentrations of eight elements—Be, Cr, Fe, Na, Pb, Ru, Sm and Zn (Table 2 and Table S5). Linking these significant effects with the data in Table S5 indicates that the shells of post-hatched, wild Capercaillie eggs tended to have higher levels of these elements compared to the eggshells of captive birds. Specifically, the concentrations of Cr, Pb, Ru and Zn in the spotted regions of the shells of captive Capercaillie eggs were 2.7–3.3-fold higher than in those of the wild birds (see Table S5).

### Changes in elemental composition between the pigment spot and background colour shell regions following embryonic eggshell etching

In both Black Grouse and Capercaillie (Fig. 1) we observed substantial embryo-induced eggshell thinning in both the background colour (− 12.0% and − 17.4%, respectively) and pigment spot regions (− 11.2% and − 16.6%, respectively) (ANOVA, in each case *P* < 0.00001; Table S1).

**Figure 3:**
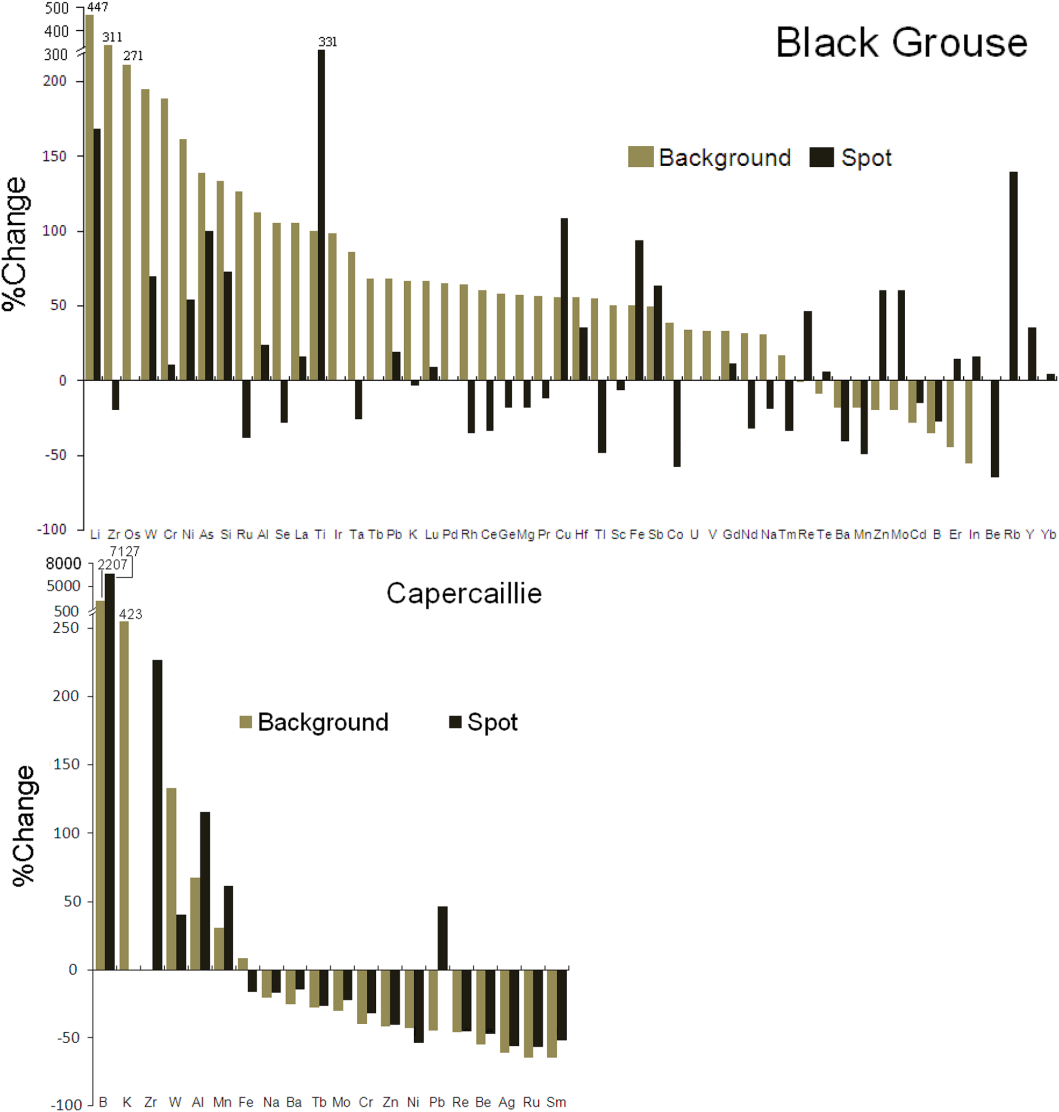
%Change (= Concentration_post-hatched_ − Concentration_non-embryonated_ × 100/Concentration_non-embryonated_) in elemental concentrations in the background colour and pigment spot regions of eggshells following embryonic eggshell etching, defined as non-embryonated and post-hatched eggshells of Black Grouse *Tetrao tetrix* and Capercaillie *Tetrao urogallus*. For the elemental concentrations and sample sizes, see Tables S2and S3. *Note*: The bars with positive values indicate elevated elemental concentrations following embryonic eggshell etching, whereas those with negative values indicate decreases within a given shell region. Note that because of some differences in eggshell elemental concentrations related to the origin of the eggs (see “Results”), the trend in %Change calculated within the sample of eggshells from wild and captive Black Grouse and Capercaillie retains the same direction, although the magnitude (bars) of %Change becomes smaller (after recalculation of the data from Tables S4 and S5).

Figure 3 shows the %Change in elemental concentrations in the background colour and pigment spot regions between non-embryonated and post-hatched eggshells, based on the data from Tables S2 and S3 (see Supplemental Material Appendix 1). As evidenced by the results of the sign test, the trends of %Change in elemental concentrations occurring in the background colour and pigment spot regions (Fig. 3) varied significantly between non-embryonated and post-hatched eggshells in both Black Grouse (Z = 7.74, *P* = 0.0061, *n* = 43) and Capercaillie (Z = 2.59, *P* = 0.0095, *n* = 18). In both species, the trends in %Change for most elemental concentrations were reversed following embryonic eggshell etching, however, the directions of the trends were not consistent for all elements and varied between the two species (Fig. 3). In detail, this is shown by the different trends in the levels of 13 elements—B, Zr, Mn, Fe, Na, Tb, Mo, Cr, Zn, Ni, Pb, Re and Ru—mostly decreasing in Capercaillie but increasing in Black Grouse (Fig. 3; Supplemental Material Appendix 1).

## Discussion

This comparative study yielded novel, quantitative ICP-OES-based ionomic data on colour-assisted within-egg variability in the elemental composition of two adjacent, i.e. pigment spot and background colour, shell regions. It identified the trend and magnitude of changes in the levels of individual chemical elements in these two differently coloured shell regions following embryonic eggshell resorption in two related gallinaceous bird species. We demonstrated empirically that females allocated many tens of chemical elements into their eggs, whose role in biological systems or animal physiology is unknown. Presumably they are distributed within specific microstructures of the mineral composition and/or different macromolecules (protein, lipids) occurring in varying amounts in the three principal shell layers. The two major results emerging from our investigation, i.e. the answers to our main research questions, are discussed below.

First, our results show an apparent local, within-egg functional disparity between the pigment spot and background colour regions of eggshells in the distribution of chemical elements at the two extremes of avian embryonic development: most elemental concentrations were higher in the speckled regions of the shell, with the differences becoming less pronounced following shell resorption. Importantly, both approaches to our analysis of eggshell ionomic data—as pairs, and over all the eggshells—generally yielded the same trends, particularly in the case of non-embryonated eggs. The most plausible explanation for the disparities in the pigment spot-background colour eggshell ionome should be derived from the presence of larger amounts of protoporphyrin (and the accompanying elemental pool) in the pigment spot region of the shell. This concurs with a previous conceptual ecophysiological framework linking the intensity of colouration with the element/metal load in animal tissues^53,54^. In this sense, our findings strongly suggest a functional role of egg speckling in the physiological deactivation of different trace elements through their incorporation into pigment spots. In the two gallinaceous species studied here, these are situated in the less calcified external shell layer, which does not participate in micronutrient resorption by developing embryos. This pattern seems to be robust throughout embryonic development, and is presumably physiologically fixed in all maculated birds’ eggs at least in Galliformes. However, one question that still needs to be addressed relates to the analogous patterns of element distributions in eggshells of other bird orders, such as Passeriformes or Falconiformes, where the pigment layer(s) is(are) embedded differently within the shell thickness cross-section^18,19,21^. Therefore, these key findings are potentially of far greater general interest, since the suggestion of new roles for pigmentation is surely of ecological and evolutionary interest and also raises questions in ecotoxicology.

From the methodological point of view, detection of such local within-egg differences can be difficult, particularly when both sample masses and eggs are small. This is because ICP-OES measures the concentrations of a large number of elements at a relatively high detection limit (> 0.01 ppm). But some trace elements measured by other ICP-MS methods can occur in avian eggshells in much lower concentrations (even < 1000-fold)^29^, which is an overall indication that the different methods of chemical analysis need to be tailored to specific research purposes.

At the same time, both species showed quite a distinct pattern in the relative differences between elemental concentrations as expressed by S/B ratios in the course of embryonic development. However, the different numbers of elements measured in the eggshells of each species (apparently attributable to the smaller mass of the Capercaillie samples used in the chemical analysis) makes interspecific comparison difficult; this is possible for just a limited number of elements. Specifically, we observed that during embryonic growth in Black Grouse, the relative differences between these two shell regions as expressed by S/B were smaller for most elements, whereas the opposite pattern prevailed in the Capercaillie samples. The trends of changes following embryonic eggshell etching in the pigment spot and background colour regions of shells were conflicting, and varied between the two species for a large group of 13 elements. In particular, we found an enormous increase in the B concentration in Capercaillie eggshells, with the trend being reversed in Black Grouse (Fig. 3). We hypothesized that a convincing working explanation for these interspecific differences could be based on the variable composition of mostly Ca and Mg, which is due to the different thicknesses of the individual shell layers, particularly as there was a relatively smaller difference in shell thickness in the pigment spot and background colour regions in the Black Grouse eggs (cf. Fig. 1); this implied that their shells were less intensively pigmented. These differences presumably translate into the varying contents of Ca and Mg in the major shell layers, especially as the S/B ratio of Mg varied in the Black Grouse eggshells (see Figs. 3 and S1). However, to resolve these conflicting results, we strongly encourage further exploration of the chemical composition of eggshell material/samples taken from shell layers containing only pigments and devoid of highly calcified mammillary bodies.

By contrast, there is a group of elements present in higher concentrations in the background colour region. However, because the analysis of the pooled data presented in Fig. 2 (derived from eggshell samples of different origin varying in concentrations of some of the elements, as revealed by the significant origin × sample × status interaction; Table 2) needs to be treated with caution, there is greater justification for basing our main conclusions on the paired data presented in Fig. S1. Consequently, the concentration of just one element (Gd, a REE, but present at a low level) measured in Black Grouse eggshells were consistently higher in the background colour than in the pigment spot region in both non-embryonated and post-hatched eggshells. As shells tend to become thinner or assimilate elements from the inner shell layer, we observed that in Black Grouse, the disparity between the levels of 13 elements (Ge, Li, Zr, Pr, K, Na, Gd, Sc, Ba, Tb, Mg, Rh, Mn) in the pigment spot and background colour regions changed (Fig. S1). This suggests, as discussed above, a higher content of these elements in the external shell layer(s), which basically contain relatively more Mg than the inner/mammillary layer, where the Ca content is higher^26,27^.

Interestingly, if we look at the results of potential environmental pollution due to trace element enrichment, and take into account the species-specific data (Tables S2and S3),we see that Capercaillie eggshells had considerably higher concentrations of 12 metals (Al, B, Cd, Fe, Mn, Na, Ni, Pb, Re, Ru, Tb and Zn) than those of Black Grouse, but that the K concentration was higher in the latter’s eggshells. In part, as mentioned above, these interspecific differences may have been due to anatomical differences in shell structure and/or pigment accumulation, to ecological differences between the diets of these species relating to diet (Capercaillie hensin particular often ingest small particles of grit from dirt roads) or to the origin of the hens. Our previous study showed that the eggs of wild Capercaillies exhibited higher Mg and Ca levels in the shells and higher levels of three heavy metals (Cu, Cd and Pb) in the contents, which suggests that these elements could have been acquired from the local mineral ingredients of the diet (grit)^28^. It is likely that under natural conditions (ground-foraging gallinaceous birds often ingest grit particles), exposure to high levels of dietary Pb or Cd may run parallel with Ca ingestion, which to some extent simultaneously reduces the bioavailability of non-essential metals^28,55,56^.

On comparing previous data regarding REE concentrations in various biological samples^37,38^, we found surprisingly high concentrations of two light REEs: Pr (Black Grouse) and Sm (Capercaillie). The concentrations of both these elements, however, exhibit different trends during embryonic development (Pr[+] and Sm[−])—they are differently distributed in the background colour and pigment spot shell regions. Because there is an apparent decrease in the Sm concentration during embryonic development, particularly in wild Capercaillie eggs, where we measured respective 4.4-fold and 3.7-fold decreases in Sm levels in these two shell regions (see Table S5), intensive Sm assimilation by embryonic tissues is a possibility. Hence, the need for further research to assess the effect of this element on embryonic development.

## Conclusions and perspectives

Our paper has revealed a wider range of rare elements, including heavy metals, in the eggshells of two endangered species than had previously been known. The question must arise as to whether these elements originated as environmental pollutants, and whether might they have contributed to the failure of some eggs to hatch, or indeed even to the decline of these species. So we need to know whether the unhatched eggs are representative of the egg sample with normal embryonic growth. For ethical reasons, however, such sampling is not possible in many wild species^13^, like Capercaillies and Black Grouse, particularly as such studies require the destructive sampling of irreplaceable collected material. This is one reason why, ethically, it is essential that the benefits of this work for the conservation of these species be brought to the fore.

Importantly, our earlier study on the levels of major micronutrients and trace elements measured in the egg contents and eggshells of Capercaillies (obtained from same areas as the eggshells analysed in this paper) suggested that there were no signs of intoxication or embryonic mortality as a result of trace element enrichment^28^. Thus, as we have already argued, most of the unhatched eggs in our previous sample of Capercaillie eggs (and most probably in the egg sample examined in this paper) must have died from resource deficiencies or incubation failures, especially as the unhatched eggs we examined previously had generally low or insufficient levels of Ca in the contents, which could have been the primary cause of infertility or embryonic mortality^28^.

In turn, one potential way of assessing the representativeness of egg samples in the ecotoxicological context would be to analyse historical samples of eggshells from museum or private collections—eggshells collected in pre-industrial times generally contain lower levels of heavy metals or pesticide residues^57,58^. However, it should be remembered that the critical requirement in such studies is the correct determination of the egg’s developmental status^9,13,15,59^. In practice, a great many eggshells in museum collections are not furnished with labels stating the status (age) of embryos, and display a variety of defects, such as egg content remnants within^3^, which may have been derived from embryonic tissues of an unknown developmental stage. This uncertainty surrounding the determination of the developmental status of eggs limits the usefulness of every historical eggshell for such investigations. Thus, if we wish to obtain unbiased results with regard to trends in historical ecotoxin/pollutant loads, only those eggshells with a specifically labelled embryonic developmental stage are suitable.

The key conclusion from this investigation is that all chemical analyses of eggshells require standardized eggshell sampling procedures in order to unify their colouration and embryonic status. We realize, however, that such an assessment of colouration is in many cases impossible, e.g. when only eggshell fragments are available. The potential ideal resolution of this problem would be (1) to analyse the entire eggshell, and (2) to perform digital imaging of the coverage of pigment spotting and background colouration on the shells, which would provide unbiased data on the contribution of these two variously coloured regions (sensu^60^), especially as the intensity of spotting is correlated with the protoporphyrin content in eggshells^61^. Nonetheless, we wish to emphasize that the protoporphyrin content in eggshells must be measured in eggshells of known developmental status, because this should be higher in post-hatched eggshells, i.e. those without mammillary bodies, through having a disproportionately thicker pigmented layer, than in unresorbed shells. Therefore, we strongly recommend further multidisciplinary investigations of the basic relationships between the colouration and chemical composition of eggshells. They should include the eggs of different bird species with variable distributions of pigment layer(s)/spots within the shell thickness, from orders with different eggshell structures, e.g. Passeriformes, Charadriiformes and Accipitriformes. We also highlight the need to measure other major chemical elements in the background colour and pigment spot regions, in particular C, N and P, which are staple elements in avian eggshells, knowledge of which is scanty, and to link these with within-egg variability in shell thickness and size-related traits of eggs.

## Supplementary Information

Supplementary Information.

## Data Availability

The datasets analysed during the current study are available from the corresponding author on reasonable request.
