# Supplementary material for: Quantifying the functional disparity in pigment spot-background egg colour ICP-OES-based eggshell ionome at two extremes of avian embryonic development
Source: Sci Rep. 2020 Dec 17;10:22107. doi: 10.1038/s41598-020-79040-4 (PMC7747640; doi:10.1038/s41598-020-79040-4)
Supplement: Supplementary file 1 — Supplementary Information. [file 41598_2020_79040_MOESM1_ESM.docx]

Supplemental Material for:

**Quantifying the functional disparity in pigment spot-background egg colour ICP-OES-based eggshell ionome at two extremes of avian embryonic development**

Grzegorz Orłowski,^1*^ Przemysław Niedzielski,^2^ Dorota Merta,^3^ Przemysław Pokorny,^4^ Jędrzej Proch^2^

^1^Institute of Agricultural and Forest Environment, Polish Academy of Sciences, Bukowska 19, 60-809 Poznań, Poland.

^2^Department of Analytical Chemistry, Adam Mickiewicz University, Umultowska 89b, 61-614 Poznań, Poland.

^3^Department of Ecology and Environmental Protection, Pedagogical University of Kraków, Podbrzezie 3, 31-054 Kraków, Poland.

^4^ Department of Limnology and Fishery, Institute of Animal Breeding, Wrocław University of Environmental and Life Sciences, Chełmońskiego 38C, 51-630 Wrocław, Poland.

* Corresponding author; e-mail: orlog@poczta.onet.pl

Appendix 1.

**Elemental composition of background colour and pigment spot regions of eggshells – in-depth analysis**

The frequency of detectability (= number of samples with concentrations exceeding the detection limit or detects) of individual eggshell elements varied strongly in both species (Table 1).

Only 17 elements (Al, Ba, Cr, Cu, Fe, K, La, Mg, Mn, Na, Nd, Pb, Pr, Sc, Si, Tb and Zn) occurred in concentrations exceeding the detection limit in each eggshell sample of the background colour and pigment spot regions of Black Grouse eggshells, while the number of non-detects for the remaining elements varied from 1 to as many as 29 (Table 1). There is no statistical difference in the detectability of the 51 elements occurring simultaneously in the eggshell samples from the background colour and pigment spot regions (Wilcoxon test, Z_51_ = 0.529, *P* = 0.567; based on data presented in Table 1).

Twelve elements (Al, Ba, Be, Cr, Fe, Mn, Na, Ni, Ru, Sm, Tb and Zn) occurred in concentrations exceeding the detection limit in each eggshell sample of the background colour and pigment spot regions of Capercaillie eggshells, while the number of non-detects for the remaining elements varied from 3 to as many as 34 (Table 1).The detectability of 25 elements, occurring simultaneously in both eggshell samples in concentrations exceeding the detection limit, was significantly higher in the background colour samples (Wilcoxon test, Z_25_ = 2.93, *P* = 0.0033).

The respective concentrations of 42 and 30 of the 45 and 43 eggshell elements measured simultaneously as a pigment spot – background colour pair within the same non-embryonated eggs and post-hatched shells of Black Grouse were higher in the pigment spot regions (Fig. S1, Table S6). This relationship in Capercaillies was even more marked, since of the 16 and 18 eggshell elements measured within the same non-embryonated eggs and post-hatched shells, only the concentrations of one simple essential element (Ba and K, respectively) was higher in the background colour region(Fig. S1, Table S7).

Visual inspection of data in Fig. 2 shows that in the case of Black Grouse, the concentrations of only two of the 46 elemental concentrations established for non-embryonated eggs (Mo, spot/background ratio = 0.909; and Gd, ratio = 0.634) were higher in the background colour region, as indicated by their ratios being <1. In contrast, the analogous distribution of ratios among post-hatched eggshells shows that the concentrations of 11 elements (Mn, Rh, Mg, Tb, Li, Ba, Sc, Na, K, Pr and Gd) were higher in the background colour region(Fig. 2: ratios between 0.957 and 0.511). All the other elemental concentrations were higher in both the non-embryonated eggs (Fig. 2: ratios from 18.06 [Tm] to 1.010 [Pr]) and the post-hatched shells (Fig. 2: ratios from 10.31 [Tm] to 1.038 [Yb]).

For Capercaillie, this relationship was even more marked, since the concentration of only one element (Ba, ratio = 0.927) among the eggshell samples from non-embryonated eggs, and the concentrations of three elements (K, Sn and Cd; ratio = 0.552, 0,484 and 0.406, respectively) among the samples of post-hatched eggshells were higher in the background colour region(Fig. 2).

**Changes in elemental composition in the pigment spot and background colourregions following embryonic eggshell etching**

Fig. 3 shows the %Change in elemental concentrations following embryonic eggshell etching in the pigment spot and background colour regions, based on the data listed in Tables S2 and S3.

**Black Grouse.** As a result of embryonic growth, 38 and 10 elemental concentrations increased (up to +467% [Li]) or decreased (down to –56% [In]), respectively (Fig. 3) in the background colour region, whereas26 and 21 elemental concentrations increased (up to +331% [Ti]) or decreased (down to –65% [Be]), respectively, in the pigment spot region (Fig. 3). The trends in %Change were not consistent (i.e. both positive and negative changes) with respect to the levels of 19 elements (Fig. 3).

**Capercaillie.** The levels of six elements (B, K, Zr, W, Al and Mn) measured in both the pigment spot and background colour regions consistently increased during embryonic growth; in particular, B concentrations increased by 2207% and 7127% in these two shell regions, respectively (Fig. 3). In contrast, the concentrations of 13 elements (Na, Ba, Tb, Mo, Cr, Zn, Ni, Pb, Re, Be, Ag, Ru and Sm) consistently decrease, down to –65% and –52%in the background colour and pigment spot regions, respectively (Fig. 3). Only the Fe concentrations displayed a different trend (Fig. 3).


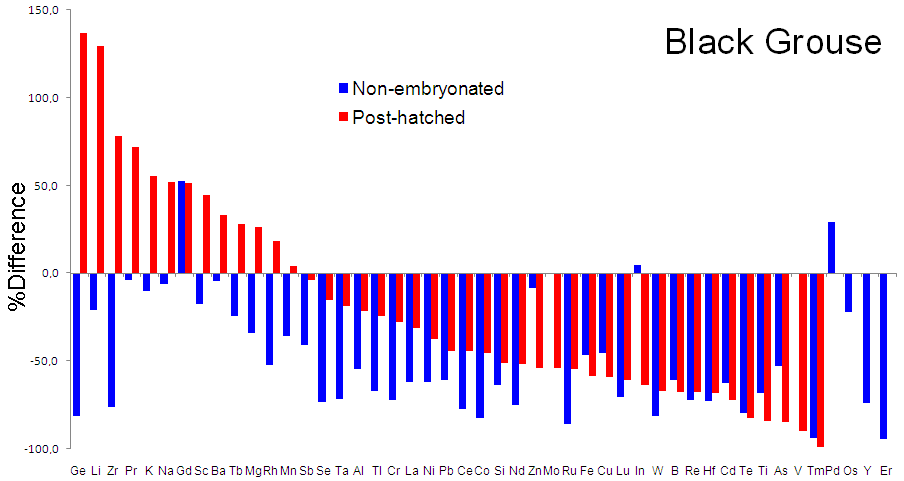


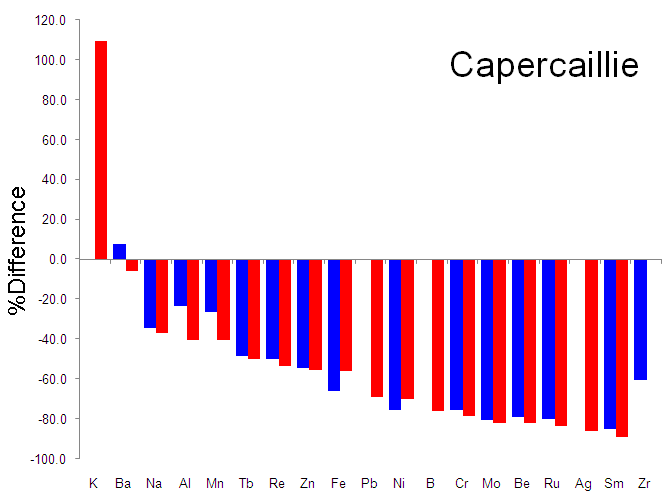


Fig S1. %Difference (= Concentration_Spot_ – Concentration_background_ × 100 / Concentration_Spot_) in elemental concentrations measured in the pigment spot-background colour shell regions in the same non-embryonated and post-hatched eggs of Black Grouse *Tetrao tetrix* and Capercaillie *Tetrao urogallus*. The corresponding elemental concentrations and *t*-test results of paired comparisons are listed in Tables S6 and S7.

*Notes:*

(1) The bars with negative values indicate higher elemental concentrations in the pigment spot region of a shell.

(2) Despite the different number of elements determined in the eggshell samples of both species (which in part seems attributable to the smaller mass of samples from Capercaillies used in the chemical analysis), the disparity in spot-background elemental composition is extremely similar in both species, because only the trends for Ba, Na, Mn and Tb vary in direction.

Table S1. T-statistics (t- and *P*-values) of eggshell thicknesses (expressed in mm) measured (with the inner shell membrane attached) in the pigment spot and adjacent background colour shell regions of all eggs, non-embryonated eggs and post-hatched shells of cryptic eggs of Black Grouse *Tetrao tetrix* and Capercaillie *Tetrao urogallus* (see Fig. 1).

| Species / Egg sample / Eggshell region | Average | ±SD | *n* | Difference | -95%CL | +95%CL | %Diff. | *t* | *df* | *P*-value† |
| --- | --- | --- | --- | --- | --- | --- | --- | --- | --- | --- |
| BLACK GROUSE | |  |  |  |  |  |  |  |  |  |
| **All eggs** |  |  |  |  |  |  |  |  |  |  |
| Background | 0.197 | 0.023 |  |  |  |  |  |  |  |  |
| Spot | 0.203 | 0.024 | 180 | -0.0054 | -0.0064 | -0.0045 | **-2.673** | -11.39 | 179 | <0.00001 |
| **Non-embryonated eggs** | |  |  |  |  |  |  |  |  |  |
| Background | 0.209 | 0.021 |  |  |  |  |  |  |  |  |
| Spot | 0.214 | 0.021 | 96 | -0.0056 | -0.0064 | -0.0047 | **-2.598** | -13.56 | 95 | <0.00001 |
| **Post-hatched eggshells** | |  |  |  |  |  |  |  |  |  |
| Background | 0.184 | 0.019 |  |  |  |  |  |  |  |  |
| Spot | 0.190 | 0.021 | 84 | -0.0053 | -0.0071 | -0.0034 | **-2.769** | -5.78 | 83 | <0.00001 |
| CAPERCAILLIE | |  |  |  |  |  |  |  |  |  |
| **All eggs** |  |  |  |  |  |  |  |  |  |  |
| Background | 0.258 | 0.037 |  |  |  |  |  |  |  |  |
| Spot | 0.272 | 0.039 | 155 | -0.0140 | -0.0154 | -0.0125 | **-5.127** | -18.92 | 154 | <0.00001 |
| **Non-embryonated eggs** | |  |  |  |  |  |  |  |  |  |
| Background | 0.299 | 0.019 |  |  |  |  |  |  |  |  |
| Spot | 0.313 | 0.019 | 34 | -0.0141 | -0.0165 | -0.0116 | **-4.496** | -11.69 | 33 | <0.00001 |
| **Post-hatched eggshells** | |  |  |  |  |  |  |  |  |  |
| Background | 0.247 | 0.033 |  |  |  |  |  |  |  |  |
| Spot | 0.261 | 0.036 | 121 | -0.0139 | -0.0157 | -0.0122 | **-5.340** | -15.75 | 120 | <0.00001 |

†In each case, the exact *P*-value is always equal to 0.

*Note:* In both species the eggshell thickness measured in the background colour and pigment spot shell regions (based on measurements averaged per egg) varied significantly among eggshells of non-embryonated eggs and among post-hatched eggshells (ANOVA, in each case *P* < 0.00001).

Table S2. Descriptive statistics of ICP-OES-based elemental concentrations (ppm d.w.) measured in the background colour and pigment spot shell regions of all non-embryonated and post-hatched eggshells of Black Grouse *Tetrao tetrix*. Light (LREEs) and heavy (HREEs) rare earth elements are designated by † and ††, respectively. BDL – all samples below the detection limit. The averages differing significantly from each other (one-way ANOVA, *P* ≤ 0.05) are boxed, and *P*-values are highlighted in red font.

| **Element** | **NON-EMBRYONATED** | | | | |  |  |  |  |  |  | **POST-HATCHED** | | | | |  | |  |  |  |  |  |
| --- | --- | --- | --- | --- | --- | --- | --- | --- | --- | --- | --- | --- | --- | --- | --- | --- | --- | --- | --- | --- | --- | --- | --- |
|  | **Background** | | | |  | **Spot** | |  |  |  |  | **Background** | | |  |  | **Spot** | | |  |  |  |  |
|  | n | average | SE | min | max | n | average | SE | min | max | *P*-value | n | average | SE | min | max | n | average | | SE | min | max | *P*-value |
| Ag | BDL | - | - | - | - | 1 | 2.092 |  |  |  |  | BDL |  |  |  |  | BDL | - | | - | - | - | - |
| Al | 16 | 10.67 | 1.110 | 3.794 | 19.873 | 16 | 23.30 | 1.716 | 10.62 | 33.51 | 0.0000 | 14 | 22.69 | 3.698 | 11.40 | 62.76 | 14 | 28.87 | | 4.55 | 7.94 | 75.71 | 0.3017 |
| As | 10 | 1.265 | 0.213 | 0.349 | 2.219 | 5 | 3.075 | 1.328 | 0.242 | 7.905 | 0.0800 | 6 | 3.016 | 1.334 | 0.360 | 8.914 | 3 | 6.157 | | 1.600 | 3.128 | 8.565 | 0.1994 |
| B | 16 | 0.340 | 0.101 | 0.031 | 1.471 | 9 | 1.324 | 0.408 | 0.040 | 2.901 | 0.0067 | 14 | 0.223 | 0.055 | 0.010 | 0.825 | 7 | 0.954 | | 0.610 | 0.025 | 4.524 | 0.1035 |
| Ba | 16 | 390.2 | 59.89 | 102.9 | 1003.1 | 16 | 407.7 | 60.21 | 110.9 | 1036.7 | 0.8382 | 14 | 319.7 | 44.84 | 81.20 | 678.4 | 14 | 240.0 | | 43.18 | 50.70 | 543.3 | 0.2119 |
| Be | BDL | - | - | - | - | 4 | 0.033 | 0.015 | 0.013 | 0.078 | - | 1 | 0.010 | - | - | - | 1 | 0.012 | |  |  |  |  |
| Bi | BDL | - | - | - | - | 1 | 178.02 | - | - | - | - | 1 | 0.120 | - | - | - | BDL | - | | - | - | - | - |
| Cd | 6 | 0.028 | 0.006 | 0.016 | 0.051 | 8 | 0.103 | 0.013 | 0.035 | 0.158 | 0.0006 | 5 | 0.023 | 0.007 | 0.011 | 0.046 | 8 | 0.087 | | 0.016 | 0.040 | 0.158 | 0.0097 |
| Ce† | 16 | 0.525 | 0.065 | 0.082 | 0.943 | 15 | 2.170 | 0.306 | 0.413 | 4.716 | 0.0000 | 14 | 0.836 | 0.189 | 0.112 | 2.296 | 12 | 1.432 | | 0.387 | 0.148 | 3.705 | 0.1605 |
| Co | 15 | 0.130 | 0.028 | 0.010 | 0.390 | 16 | 0.695 | 0.140 | 0.160 | 2.460 | 0.0006 | 12 | 0.178 | 0.033 | 0.030 | 0.490 | 13 | 0.295 | | 0.100 | 0.017 | 1.253 | 0.2971 |
| Cr | 16 | 0.097 | 0.009 | 0.050 | 0.169 | 16 | 0.347 | 0.051 | 0.102 | 0.742 | 0.0000 | 14 | 0.278 | 0.049 | 0.066 | 0.595 | 14 | 0.383 | | 0.103 | 0.050 | 1.261 | 0.3665 |
| Cu | 16 | 1.251 | 0.113 | 0.611 | 2.191 | 16 | 2.291 | 0.317 | 0.861 | 5.239 | 0.0043 | 14 | 1.955 | 0.328 | 0.880 | 5.567 | 14 | 4.783 | | 1.514 | 0.936 | 22.18 | 0.0795 |
| Er†† | 2 | 0.018 | 0.007 | 0.010 | 0.025 | 4 | 0.120 | 0.059 | 0.023 | 0.281 | 0.3155 | 1 | 0.014 |  |  |  | 7 | 0.137 | | 0.060 | 0.026 | 0.477 | 0.4955 |
| Fe | 16 | 19.63 | 4.078 | 6.956 | 69.08 | 16 | 36.64 | 9.149 | 11.842 | 142.30 | 0.0998 | 14 | 29.44 | 5.732 | 11.076 | 73.66 | 14 | 70.86 | | 27.96 | 9.905 | 352.4 | 0.1586 |
| Ga | BDL | - | - | - | - | 1 | 0.137 | - | - | - | - | BDL | - | - | - | - | BDL | - | | - | - | - | - |
| Gd† | 15 | 0.090 | 0.007 | 0.052 | 0.154 | 4 | 0.057 | 0.012 | 0.023 | 0.079 | 0.0452 | 14 | 0.124 | 0.028 | 0.018 | 0.420 | 3 | 0.064 | | 0.047 | 0.013 | 0.157 | 0.3587 |
| Ge | 9 | 0.744 | 0.213 | 0.056 | 1.719 | 2 | 1.911 | 0.543 | 1.368 | 2.453 | 0.0488 | 9 | 1.172 | 0.496 | 0.032 | 4.789 | 7 | 1.561 | | 0.920 | 0.020 | 6.482 | 0.6984 |
| Hf | 15 | 0.356 | 0.044 | 0.126 | 0.703 | 15 | 1.298 | 0.211 | 0.126 | 2.854 | 0.0002 | 14 | 0.556 | 0.141 | 0.031 | 2.157 | 14 | 1.753 | | 0.372 | 0.224 | 4.850 | 0.0058 |
| Hg | 2 | 0.017 | 0.002 | 0.015 | 0.019 | BDL | - | - | - | - | - | BDL | - | - | - | - | 1 | 0.04 | | - | - | - | - |
| Ho†† | 2 | 0.015 | 0.001 | 0.015 | 0.016 | BDL | - | - | - | - | - | BDL | - | - | - | - | BDL | - | | - | - | - | - |
| In | 6 | 1.200 | 0.272 | 0.062 | 1.938 | 5 | 2.833 | 1.473 | 0.335 | 8.193 | 0.2617 | 4 | 0.531 | 0.257 | 0.093 | 1.218 | 7 | 3.277 | | 0.865 | 0.696 | 7.433 | 0.0458 |
| Ir | 3 | 0.686 | 0.416 | 0.012 | 1.445 | BDL | - | - | - | - | - | 6 | 1.367 | 0.755 | 0.265 | 5.107 | BDL | - | | - | - | - | - |
| K | 16 | 918.2 | 32.76 | 737.3 | 1191.4 | 16 | 1020.52 | 41.30 | 564.37 | 1276.7 | 0.0617 | 14 | 1530.6 | 265.1 | 817.2 | 3869.8 | 14 | 983.8 | | 108.9 | 252.1 | 1587.7 | 0.0675 |
| La† | 16 | 0.188 | 0.027 | 0.089 | 0.474 | 16 | 0.483 | 0.079 | 0.169 | 1.482 | 0.0013 | 14 | 0.385 | 0.086 | 0.081 | 1.065 | 14 | 0.558 | | 0.096 | 0.085 | 1.261 | 0.1911 |
| Li | 15 | 0.086 | 0.011 | 0.010 | 0.141 | 8 | 0.142 | 0.034 | 0.036 | 0.346 | 0.0639 | 13 | 0.506 | 0.357 | 0.024 | 4.766 | 7 | 0.382 | | 0.204 | 0.023 | 1.530 | 0.8127 |
| Lu†† | 15 | 0.063 | 0.005 | 0.028 | 0.097 | 16 | 0.228 | 0.044 | 0.011 | 0.815 | 0.0011 | 14 | 0.098 | 0.026 | 0.033 | 0.376 | 14 | 0.248 | | 0.056 | 0.062 | 0.731 | 0.0221 |
| Mg | 16 | 2449.5 | 137.842 | 1575.4 | 3446.0 | 16.0 | 3715.7 | 155.7 | 2592.2 | 4870.1 | 0.0000 | 14 | 3841.2 | 710.3 | 1699.0 | 9568.1 | 14 | 3042.1 | | 307.5 | 1018.9 | 4585.8 | 0.3114 |
| Mn | 16 | 11.399 | 2.854 | 1.265 | 44.648 | 16 | 17.75 | 3.989 | 2.07 | 55.92 | 0.2050 | 14 | 9.325 | 1.741 | 1.825 | 24.445 | 14 | 8.92 | | 2.02 | 1.03 | 22.68 | 0.8816 |
| Mo | 6 | 0.147 | 0.031 | 0.032 | 0.260 | 2 | 0.134 | 0.121 | 0.012 | 0.255 | 0.8720 | 4 | 0.120 | 0.035 | 0.035 | 0.201 | 5 | 0.21 | | 0.05 | 0.03 | 0.30 | 0.1852 |
| Na | 16 | 1110.3 | 27.847 | 923.2 | 1270.7 | 16.0 | 1179.4 | 33.493 | 889.9 | 1371.1 | 0.1232 | 14 | 1448.8 | 232.259 | 831.844 | 3469.7 | 14 | 951.1 | | 110.6 | 292.4 | 1847.4 | 0.0640 |
| Nb | BDL | - | - | - | - | BDL |  |  |  |  |  | 1 | 0.010 | - | - | - | BDL | - | | - | - | - | - |
| Nd† | 16 | 1.090 | 0.123 | 0.265 | 1.993 | 16 | 4.352 | 0.605 | 1.567 | 11.923 | 0.0000 | 14 | 1.428 | 0.238 | 0.256 | 3.510 | 14 | 2.951 | | 0.466 | 0.842 | 7.329 | 0.0073 |
| Ni | 14 | 0.310 | 0.040 | 0.118 | 0.630 | 14 | 0.843 | 0.113 | 0.158 | 1.564 | 0.0001 | 12 | 0.811 | 0.147 | 0.365 | 1.786 | 13 | 1.297 | | 0.500 | 0.140 | 6.938 | 0.3769 |
| Os | 1 | 0.068 |  | 0.068 | 0.068 | 3 | 0.183 | 0.073 | 0.090 | 0.326 | 0.5086 | 3 | 0.263 | 0.155 | 0.033 | 0.559 | BDL | - | | - | - | - | - |
| Pb | 16 | 1.696 | 0.150 | 0.456 | 2.555 | 16 | 4.313 | 0.570 | 0.055 | 9.122 | 0.0001 | 14 | 2.855 | 0.584 | 1.233 | 7.170 | 14 | 5.122 | | 1.060 | 1.203 | 15.91 | 0.0723 |
| Pd | 9 | 0.169 | 0.029 | 0.063 | 0.313 | 1 | 0.171 |  | 0.171 | 0.171 | 0.9851 | 9 | 0.280 | 0.097 | 0.015 | 0.805 | BDL | - | | - | - | - | - |
| Pr† | 16 | 34.93 | 0.532 | 31.97 | 40.29 | 16 | 36.19 | 0.853 | 27.04 | 41.09 | 0.2195 | 14 | 54.60 | 10.10 | 33.88 | 136.5 | 14 | 31.78 | | 3.21 | 9.09 | 43.65 | 0.0407 |
| Rb | BDL | - | - | - | - | 6 | 21.08 | 5.732 | 4.446 | 45.02 | - | BDL | - | - | - | - | 3 | 50.50 | | 47.00 | 2.38 | 144.50 | - |
| Re | 9 | 0.354 | 0.099 | 0.040 | 0.926 | 10 | 0.985 | 0.286 | 0.015 | 3.010 | 0.0629 | 10 | 0.353 | 0.052 | 0.124 | 0.640 | 11 | 1.437 | | 0.447 | 0.051 | 4.218 | 0.0332 |
| Rh | 16 | 0.255 | 0.032 | 0.043 | 0.633 | 14 | 0.539 | 0.100 | 0.052 | 1.398 | 0.0080 | 14 | 0.415 | 0.060 | 0.038 | 0.886 | 14 | 0.350 | | 0.087 | 0.088 | 1.342 | 0.5452 |
| Ru | 14 | 0.151 | 0.027 | 0.015 | 0.385 | 14 | 1.038 | 0.243 | 0.435 | 3.870 | 0.0012 | 12 | 0.344 | 0.107 | 0.066 | 1.307 | 12 | 0.637 | | 0.177 | 0.061 | 1.740 | 0.1701 |
| Sb | 11 | 14.97 | 2.817 | 5.225 | 32.34 | 10 | 22.55 | 5.964 | 0.047 | 53.73 | 0.2508 | 8 | 22.405 | 5.816 | 3.544 | 47.16 | 8 | 36.84 | | 14.74 | 0.747 | 104.2 | 0.3777 |
| Sc†† | 16 | 0.099 | 0.009 | 0.073 | 0.229 | 16 | 0.114 | 0.007 | 0.029 | 0.150 | 0.1887 | 14 | 0.155 | 0.027 | 0.080 | 0.352 | 14 | 0.107 | | 0.011 | 0.024 | 0.171 | 0.1112 |
| Se | 15 | 0.909 | 0.134 | 0.272 | 1.938 | 12 | 3.192 | 0.800 | 0.053 | 8.171 | 0.0043 | 12 | 1.865 | 0.414 | 0.168 | 4.214 | 11 | 2.275 | | 0.867 | 0.338 | 9.846 | 0.6659 |
| Si | 16 | 7.888 | 0.802 | 2.926 | 13.27 | 16 | 21.686 | 2.304 | 6.631 | 38.932 | 0.0000 | 14 | 18.393 | 3.016 | 7.696 | 48.91 | 14 | 37.380 | | 13.06 | 5.352 | 189.6 | 0.1684 |
| Ta | 11 | 0.205 | 0.058 | 0.019 | 0.698 | 8 | 0.795 | 0.143 | 0.101 | 1.399 | 0.0006 | 14 | 0.387 | 0.163 | 0.019 | 2.416 | 9 | 0.589 | | 0.165 | 0.129 | 1.492 | 0.4135 |
| Tb†† | 16 | 0.467 | 0.015 | 0.342 | 0.551 | 16 | 0.616 | 0.053 | 0.207 | 0.986 | 0.0120 | 14 | 0.792 | 0.142 | 0.433 | 2.089 | 14 | 0.618 | | 0.083 | 0.080 | 1.122 | 0.3000 |
| Te | 16 | 1.256 | 0.146 | 0.340 | 2.168 | 15 | 6.154 | 0.986 | 2.358 | 16.03 | 0.0000 | 14 | 1.149 | 0.247 | 0.270 | 3.508 | 14 | 6.515 | | 1.713 | 0.827 | 20.503 | 0.0046 |
| Ti | 8 | 0.074 | 0.016 | 0.016 | 0.158 | 14 | 0.205 | 0.050 | 0.018 | 0.732 | 0.0697 | 12 | 0.139 | 0.045 | 0.015 | 0.565 | 14 | 0.882 | | 0.544 | 0.065 | 7.802 | 0.2206 |
| Tl | 14 | 0.583 | 0.125 | 0.032 | 1.427 | 11 | 2.151 | 0.425 | 0.213 | 4.986 | 0.0007 | 11 | 0.901 | 0.134 | 0.346 | 1.609 | 11 | 1.102 | | 0.333 | 0.157 | 3.795 | 0.5806 |
| Tm†† | 8 | 0.063 | 0.017 | 0.013 | 0.139 | 14 | 1.145 | 0.159 | 0.216 | 2.100 | 0.0001 | 3 | 0.074 | 0.053 | 0.018 | 0.179 | 13 | 0.760 | | 0.164 | 0.033 | 1.663 | 0.0709 |
| U | 8 | 0.468 | 0.108 | 0.117 | 1.127 | BDL | - | - | - | - | - | 5 | 0.631 | 0.162 | 0.211 | 1.088 | BDL | - | | - | - | - | - |
| V | 5 | 0.027 | 0.008 | 0.012 | 0.055 | BDL | - | - | - | - | - | 8 | 0.042 | 0.014 | 0.013 | 0.137 | 2 | 0.204 | | 0.185 | 0.019 | 0.389 | 0.0753 |
| W | 4 | 0.182 | 0.063 | 0.030 | 0.323 | 5 | 0.760 | 0.338 | 0.038 | 1.734 | 0.1789 | 5 | 0.527 | 0.197 | 0.275 | 1.308 | 6 | 1.287 | | 0.487 | 0.034 | 3.065 | 0.2130 |
| Y†† | 3 | 0.019 | 0.006 | 0.012 | 0.031 | 8 | 0.081 | 0.017 | 0.029 | 0.182 | 0.0643 | BDL | - | - | - | - | 5 | 0.110 | | 0.050 | 0.011 | 0.254 | - |
| Yb†† | BDL | - | - | - | - | 8 | 0.028 | 0.005 | 0.013 | 0.056 | - | 1 | 0.029 |  | 0.029 | 0.029 | 8 | 0.030 | | 0.008 | 0.011 | 0.078 | 0.9638 |
| Zn | 16 | 5.235 | 0.789 | 1.551 | 13.91 | 16 | 5.726 | 0.766 | 2.260 | 13.065 | 0.6579 | 14 | 4.212 | 1.343 | 1.545 | 20.979 | 14 | 9.156 | | 4.255 | 0.717 | 60.200 | 0.2780 |
| Zr | 3 | 0.086 | 0.026 | 0.259 | 0.036 | 10 | 0.622 | 0.174 | 0.133 | 2.112 | 0.1312 | 3 | 0.374 | 0.280 | 0.055 | 0.932 | 10 | 0.500 | | 0.133 | 0.057 | 1.303 | 0.6666 |

Table S3. Descriptive statistics of ICP-OES-based elemental concentrations (ppm d.w.) measured in the background colour and pigment spot regions of the shells of all non-embryonated eggs and post-hatched eggshells of Capercaillie *Tetrao urogallus*. Light (LREEs) and heavy (HREEs) rare earth elements are designated by † and ††, respectively. BDL – all samples below the detection limit. The averages differing significantly from each other (one-way ANOVA, *P* ≤ 0.05) are boxed, and *P*-values are highlighted in red font.

| **Element** | **NON-EMBRYONATED** | | | | |  |  |  |  |  |  | **POST-HATCHED** | | | | | |  |  |  |  |  |
| --- | --- | --- | --- | --- | --- | --- | --- | --- | --- | --- | --- | --- | --- | --- | --- | --- | --- | --- | --- | --- | --- | --- |
|  | **Background** | | | |  | **Spot** | |  |  |  |  | **Background** | | | |  | **Spot** | |  |  |  |  |
|  | n | average | ±SE | min | max | n | average | ±SE | min | max | *P*-value | n | average | ±SE | min | max | n | average | ±SE | min | max | *P*-value |
| Ag | 3 | 0.187 | 0.107 | 0.021 | 0.387 | 4 | 0.671 | 0.330 | 0.043 | 1.542 | 0.2807 | 6 | 0.073 | 0.024 | 0.020 | 0.172 | 6 | 0.294 | 0.088 | 0.014 | 0.528 | 0.0357 |
| Al | 8 | 13.68 | 1.319 | 9.921 | 19.44 | 8 | 17.85 | 4.074 | 4.254 | 33.83 | 0.3463 | 27 | 22.94 | 2.335 | 9.173 | 75.436 | 27 | 38.46 | 3.529 | 7.113 | 80.85 | 0.0006 |
| As | BDL | - | - | - | - | BDL | - | - | - | - | - | 2 | 2.148 | 0.645 | 1.503 | 2.794 | BDL | - | - | - | - | - |
| B | 3 | 0.276 | 0.137 | 0.119 | 0.549 | 2 | 0.357 | 0.242 | 0.115 | 0.598 | 0.7697 | 24 | 6.366 | 1.216 | 0.833 | 19.88 | 25 | 25.80 | 4.881 | 1.593 | 91.86 | 0.0004 |
| Ba | 8 | 165.0 | 20.74 | 90.11 | 241.1 | 8 | 152.9 | 32.11 | 31.88 | 288.6 | 0.7573 | 27 | 122.9 | 18.09 | 44.65 | 414.0 | 27 | 130.4 | 17.43 | 52.16 | 402.8 | 0.7680 |
| Be | 8 | 0.127 | 0.011 | 0.090 | 0.185 | 8 | 0.609 | 0.144 | 0.102 | 1.412 | 0.0048 | 27 | 0.057 | 0.007 | 0.018 | 0.203 | 27 | 0.321 | 0.039 | 0.113 | 0.841 | 0.0000 |
| Cd | BDL | - | - | - | - | BDL | - | - | - | - | - | 1 | 10.38 | - | 10.383 | 10.38 | 1 | 4.212 |  | 4.212 | 4.212 |  |
| Cr | 8 | 0.890 | 0.131 | 0.588 | 1.674 | 8 | 3.659 | 0.823 | 0.481 | 7.728 | 0.0051 | 27 | 0.533 | 0.089 | 0.182 | 2.496 | 27 | 2.488 | 0.435 | 0.622 | 11.55 | 0.0001 |
| Dy†† | BDL | - | - | - | - | BDL | - | - | - | - | - | 9 | 0.105 | 0.025 | 0.028 | 0.266 | BDL | - | - | - | - | - |
| Fe | 8 | 20.81 | 3.245 | 7.427 | 36.12 | 8 | 61.46 | 17.17 | 11.48 | 166.53 | 0.0355 | 27 | 22.55 | 1.930 | 7.963 | 52.19 | 27 | 51.26 | 4.430 | 18.61 | 95.63 | 0.0000 |
| K | 3 | 114.6 | 50.92 | 54.37 | 215.8 | BDL | - | - | - | - | - | 27 | 598.9 | 40.07 | 284.2 | 1412.8 | 11 | 330.7 | 45.51 | 128.7 | 581.1 | 0.0004 |
| Mn | 8 | 4.843 | 1.259 | 0.930 | 11.12 | 8 | 6.604 | 1.919 | 0.963 | 14.70 | 0.4558 | 27 | 6.344 | 0.805 | 1.479 | 17.97 | 27 | 10.66 | 2.189 | 1.955 | 56.32 | 0.0701 |
| Mo | 7 | 1.461 | 0.268 | 0.803 | 2.680 | 8 | 7.044 | 1.695 | 1.099 | 13.04 | 0.0095 | 25 | 1.020 | 0.324 | 0.022 | 8.316 | 24 | 5.451 | 2.220 | 0.063 | 52.74 | 0.0496 |
| Na | 8 | 2610.2 | 75.5 | 2392.6 | 3042.9 | 8 | 3984.3 | 701.3 | 695.9 | 7090.5 | 0.0717 | 27 | 2072.6 | 90.64 | 1126.8 | 3265.6 | 27 | 3302.8 | 191.1 | 1978.8 | 5259.6 | 0.0000 |
| Ni | 8 | 7.267 | 1.324 | 0.367 | 12.35 | 8 | 29.91 | 7.626 | 4.052 | 64.94 | 0.0111 | 27 | 4.153 | 0.733 | 0.763 | 21.461 | 27 | 13.86 | 1.703 | 1.771 | 35.54 | 0.0000 |
| Pb | 4 | 4.061 | 0.953 | 1.412 | 5.950 | 1 | 5.375 |  | 5.375 | 5.375 | 0.5812 | 11 | 2.232 | 0.477 | 0.313 | 4.940 | 8 | 7.873 | 2.432 | 0.607 | 18.81 | 0.0168 |
| Re | 6 | 3.844 | 1.259 | 1.160 | 8.741 | 3 | 7.314 | 2.230 | 3.405 | 11.13 | 0.1835 | 21 | 2.070 | 0.342 | 0.186 | 5.951 | 21 | 4.015 | 0.642 | 0.098 | 10.85 | 0.0109 |
| Ru | 8 | 4.521 | 0.449 | 2.706 | 6.328 | 8 | 22.66 | 6.889 | 3.922 | 55.15 | 0.0199 | 27 | 1.588 | 0.179 | 0.415 | 4.927 | 27 | 9.721 | 1.330 | 2.609 | 29.20 | 0.0000 |
| Sb | BDL | - | - | - | - | BDL | - | - | - | - | - | 1 | 12.60 | - | - | - | 1 | 177.7 | - | - | - | - |
| Sm† | 8 | 16.91 | 2.042 | 9.659 | 26.90 | 8 | 113.5 | 29.51 | 18.40 | 277.8 | 0.0056 | 27 | 5.935 | 1.155 | 1.020 | 29.36 | 27 | 54.45 | 7.850 | 13.310 | 156.8 | 0.0000 |
| Sn | BDL | - | - | - | - | BDL | - | - | - | - | - | 21 | 22.77 | 1.773 | 5.537 | 36.60 | 1 | 11.03 | - | - | - | - |
| Ta | BDL | - | - | - | - | BDL | - | - | - | - | - | 1 | 22.38 | - | - | - | 1 | 155.3 | - | - | - | - |
| Tb† | 8 | 1.191 | 0.120 | 0.688 | 1.723 | 8 | 2.322 | 0.557 | 0.613 | 5.670 | 0.0672 | 27 | 0.857 | 0.063 | 0.373 | 2.143 | 27 | 1.706 | 0.129 | 0.873 | 3.468 | 0.0000 |
| Th | BDL | - | - | - | - | BDL | - | - | - | - | - | 12 | 0.826 | 0.204 | 0.017 | 1.802 | BDL | - | - | - | - | - |
| Ti | BDL | - | - | - | - | 1 | 0.104 | - | - | - | - | BDL | - | - | - | - | BDL | - | - | - | - | - |
| W | 3 | 0.693 | 0.127 | 0.488 | 0.925 | 1 | 2.066 | - | - | - | - | 7 | 1.614 | 0.578 | 0.224 | 4.364 | 3 | 2.903 | 1.730 | 0.727 | 6.320 | 0.3773 |
| Zn | 8 | 11.92 | 0.951 | 8.901 | 17.15 | 8 | 26.21 | 5.156 | 5.516 | 53.33 | 0.0164 | 27 | 6.942 | 1.055 | 1.422 | 22.56 | 27 | 15.58 | 2.255 | 4.530 | 48.04 | 0.0011 |
| Zr | 3 | 1.478 | 0.782 | 0.395 | 2.996 | 3 | 3.572 | 1.648 | 0.448 | 6.045 | 0.3150 | BDL | - | - | - | - | 3 | 11.67 | 4.633 | 2.403 | 16.38 |  |

Table S4. Average ± SE (sample size) ICP-OES-based elemental concentrations (ppm d.w.) measured in the background colour and pigment spot regions of the shells of non-embryonated eggs and post-hatched eggshells of wild and captive Black Grouse *Tetrao tetrix*. BDL – all samples below the detection limit. The averages in bold, differing significantly from each other (one-way ANOVA, *P* ≤ 0.05), are boxed.

| Element | Wild Black Grouses | |  | | | |  |  | Captive Black Grouses | |  | | |  | |
| --- | --- | --- | --- | --- | --- | --- | --- | --- | --- | --- | --- | --- | --- | --- | --- |
|  | Non-embryonated | | |  | Post-hatched | | |  | Non-embryonated | | |  | Post-hatched | | |
|  | Background (11) | Spot (11) | |  | Background (4) | Spot (4) | |  | Background (5) | Spot (5) | |  | Background (10) | | Spot (10) |
| Ag | BDL | 2.09 (1) | | | BDL | BDL | | | BDL | BDL | | | BDL | | BDL |
| Al | **9.20±0.95** (11) | **20.68±1.91** (11) | | | **13.18±1.08**(4)  (4) | **34.50±3.64** (4) | | | **13.90±2.46** (5) | **29.05±1.81** (5) | | | 26.50±4.68 (10) | | 26.62±6.17 (10) |
| As | **1.32±0.23** (8) | **4.53±1.76** (3) | | | 0.75±0.39 (2) | 6.78 (1) | | | 1.05±0.70(2) | 0.90±0.65(2) | | | 4.15±1.77(4) | | 5.85±2.72(2) |
| B | **0.46±0.13** (11) | **1.90±0.45** (6) | | | 0.42±0.14 (4) | 2.05±1.24 (3) | | | 0.08±0.02 (5) | 0.17±0.07 (3) | | | 0.14±0.03 (10) | | 0.13±0.08 (4) |
| Ba | 486.2±68.11 (11) | 502.5±69.60  (11) | | | 382.1±64.25  (4) | 396.4±57.90  (4) | | | 179.1±36.18 (5) | 199.1±32.34 (5) | | | 294.7±57.20 (10) | | 177.5±42.44 (10) |
| Be | BDL | 0.040±0.02 (3) | | | BDL | BDL | | | BDL | 0.013 (1) | | | 0.010 (1) | | 0.012 (1) |
| Bi | BDL | 178.0 (1) | | | BDL | BDL | | | BDL | BDL | | | 0.120 (1) | | BDL |
| Cd | **0.029±0.007** (5) | **0.100±0.015** (7) | | | 0.018±0.005 (3) | 0.102±0.031 (3) | | | 0.024 (1) | 0.119 (1) | | | 0.029±0.017 (2) | | 0.079±0.018 (5) |
| Ce | **0.51±0.083** (11) | **2.06±0.290** (10) | | | 0.47±0.184 (4) | 1.23±0.904 (3) | | | **0.55±0.114** (5) | **2.40±0.766** (5) | | | 0.98±0.244 (10) | | 1.50±0.452 (9) |
| Co | **0.125±0.028** (10) | **0.761±0.197** (11) | | | 0.134±0.030 (3) | 0.088±0.047 (3) | | | **0.140±0.070** (5) | **0.551±0.124** (5) | | | 0.193±0.042 (9) | | 0.357±0.124 (10) |
| Cr | **0.086±0.009** (11) | **0.364±0.066** (11) | | | 0.270±0.107 (4) | 0.751±0.277 (4) | | | 0.120±0.015 (5) | 0.311±0.081 (5) | | | 0.281±0.057 (10) | | 0.236±0.053 (10) |
| Cu | **1.19±0.146** (11) | **2.05±0.369** (11) | | | 1.72±0.307 (4) | 4.01±1.074 (4) | | | 1.39±0.167 (5) | 2.81±0.603 (5) | | | 2.05±0.449 (10) | | 5.09±2.109 (10) |
| Er | 0.018±0.007 (2) | 0.066±0.036 (3) | | | BDL | 0.193±0.142 (3) | | | BDL | 0.281 (1) | | | 0.014 (1) | | 0.095±0.036 (4) |
| Fe | 18.27±5.34 (11) | 29.70±8.40 (11) | | | 36.32±14.99 (4) | 181.7±76.67 (4) | | | 22.62±6.28 (5) | 51.90±23.07 (5) | | | 26.69±5.79 (10) | | 26.54±5.72 (10) |
| Ga | BDL | 0.137(1) | | | BDL | BDL | | | BDL | BDL | | | BDL | | BDL |
| Gd | 0.092±0.010 (10) | 0.057±0.012 (4) | | | 0.071±0.020 (4) | 0.089±0.068 (2) | | | 0.086±0.009  (5) | BDL | | | 0.146±0.036 (10) | | 0.013 (1) |
| Ge | 0.740±0.245 (6) | 1.37 (1) | | | 0.59±0.022 (2) | 2.53±1.493 (4) | | | 0.75±0.497 (3) | 2.45 (1) | | | 1.34±0.633 (7) | | 0.27±0.126 (3) |
| Hf | **0.344±0.054** (10) | **1.513±0.265** (10) | | | **0.376±0.127** (4) | **2.926±1.004**(4)  (4) | | | 0.380±0.081 (5) | 0.868±0.282 (5) | | | **0.627±0.190**  (10) | | **1.283±0.246** (10) |
| Hg | 0.017±0.002 (2) | BDL | | | BDL | 0.044 (1) | | | BDL | BDL | | | BDL | | BDL |
| Ho | 0.015±0.001 (2) | BDL | | | BDL | BDL | | | BDL | BDL | | | BDL | | BDL |
| In | 1.36±0.218 (4) | 2.08±1.748 (2) | | | 1.22 (1) | 3.66 (1) | | | 0.879±0.817 (2) | 3.33±2.43 (3) | | | 0.302±0.165 (3) | | 3.21±1.02 (6) |
| Ir | 0.602 (1) | BDL | | | 0.697 (1) | BDL | | | 0.728±0.716 (2) | BDL | | | 1.502±0.910 (5) | | BDL |
| K | 895.2±36.25  (11) | 987.0±55.8  (11) | | | 1072.7±92.5 (4) | 1189.5±161.3(4) | | | 968.7±68.8 (5) | 1094.2±37.4(5) | | | **1713.8±357.7**(10) | | **901.5±133.8**(10) |
| La | **0.174±0.032**(11) | **0.529±0.103**(11) | | | **0.214±0.064**(4) | **0.776±0.178**(4) | | | 0.219±0.051(5) | 0.381±0.114(5) | | | 0.454±0.112(10) | | 0.471±0.106(10) |
| Li | 0.086±0.013(10) | 0.143±0.046(6) | | | 0.053±0.006(4) | BDL | | | 0.087±0.021(5) | 0.139±0.022(2) | | | 0.707±0.509(9) | | 0.382±0.204(7) |
| Lu | **0.066±0.006**(10) | **0.258±0.059**(11) | | | **0.047±0.005**(4) | **0.430±0.155**(4) | | | **0.058±0.012**(5) | **0.163±0.043**(5) | | | 0.118±0.035(10) | | 0.176±0.033(10) |
| Mg | **2490.9±175.3**(11) | **3837.1±209.9**(11) | | | **2531.4±125.0**(4) | **3961.0±231.4**(4) | | | **2358.5±236.6**(5) | **3448.6±150.9**(5) | | | 4365.1±954.7(10) | | 2674.6±362.7(10) |
| Mn | 14.45±3.82(11) | 22.12±5.30(11) | | | **12.36±1.87**(4) | **19.36±1.90**(4) | | | 4.69±1.12(5) | 8.16±1.70(5) | | | 8.11±2.26(10) | | 4.75±0.98(10) |
| Mo | 0.134±0.067(3) | 0.012(1) | | | **0.069±0.033**(2) | **0.269±0.030**(2) | | | 0.160±0.004(3) | 0.255(1) | | | 0.172±0.029(2) | | 0.178±0.079(3) |
| Na | 1144.7±28.8(11) | 1164.8±41.1(11) | | | 1200.5±68.3(4) | 1335.4±180.3(4) | | | 1034.6±51.6(5) | 1211.3±61.6(5) | | | **1548.1±323.5**(10) | | **797.4±105.8**(10) |
| Nb | BDL | BDL | | | BDL | BDL | | | BDL | BDL | | | 0.010 (1) | | BDL |
| Nd | **1.18±0.130**(11) | **4.69±0.852**(11) | | | **0.91±0.299**(4) | **4.54±0.96**(4) | | | **0.89±0.274**(5) | **3.62±0.450**(5) | | | 1.63±0.293(10) | | 2.31±0.398(10) |
| Ni | **0.329±0.061**(9) | **0.884±0.136**(9) | | | 0.627±0.142(4) | 2.62±1.475(4) | | | 0.277±0.033(5) | 0.771±0.216(5) | | | 0.903±0.208(8) | | 0.709±0.195(9) |
| Os | 0.068(2) | 0.112±0.022(2) | | | 0.033(1) | BDL | | | BDL | 0.326(1) | | | 0.378±0.181(2) | | BDL |
| Pb | 1.90±0.153(11) | 4.69±0.690(11) | | | 1.85±0.25(4) | 8.73±2.93(4) | | | 1.25±0.264(5) | 3.48±1.018(5) | | | 3.26±0.787(10) | | 3.68±0.561(10) |
| Pd | 0.179±0.031(8) | 0.171(1) | | | 0.046(1) | BDL | | | 0.083(1) | BDL | | | 0.309±0.104(8) | | BDL |
| Pr | 34.6±0.45(11) | 35.7±1.13(11) | | | 35.4±0.66(4) | 37.2±1.88(4) | | | 35.6±1.45(5) | 37.3±1.13(5) | | | **62.3±13.53**(10) | | **29.6±4.30**(10) |
| Rb | BDL | 20.7±7.0(5) | | | BDL | 74.6±69.9(2) | | | BDL | 23.0(1) | | | BDL | | 2.4(1) |
| Re | 0.308±0.114(7) | 0.893±0.457(6) | | | **0.262±0.066**(4) | **3.037±0.842**(2) | | | 0.516±0.227(2) | 1.121±0.285(4) | | | 0.413±0.067(6) | | 1.082±0.445(9) |
| Rh | **0.232±0.028**(11) | **0.545±0.104**(9) | | | 0.324±0.043(4) | 0.570±0.276(4) | | | 0.304±0.086(5) | 0.529±0.228(5) | | | 0.451±0.081(10) | | 0.262±0.046(10) |
| Ru | **0.141±0.034**(10) | **1.225±0.325**(10) | | | 0.209±0.058(4) | 0.998±0.322(4) | | | **0.176±0.039**(4) | **0.572±0.078**(4) | | | 0.411±0.156(8) | | 0.457±0.195(8) |
| Sb | 16.52±3.24(9) | 20.09±8.45(7) | | | 24.67(1) | 41.97±35.33(2) | | | **7.98±0.12**(2) | **28.28±3.21**(3) | | | 22.08±6.71(7) | | 35.13±17.89(6) |
| Sc | 0.102±0.013 (11) | 0.109±0.010 (11) | | | 0.123±0.026 (4) | 0.137±0.010 (4) | | | **0.092±0.006** (5) | **0.127±0.004** (5) | | | 0.168±0.036 (10) | | 0.095±0.014 (10) |
| Se | **1.03±0.169** (11) | **3.44±1.113** (8) | | | 0.853±0.179 (3) | 4.91±2.74 (3) | | | 0.586±0.058 (4) | 2.71±1.07 (4) | | | 2.203±0.504 (9) | | 1.29± 0.405 (8) |
| Si | **7.90±0.949** (11) | **19.80±2.48** (11) | | | 14.30±1.89 (4) | 46.72±16.43 (4) | | | **7.86±1.66** (5) | **25.84±4.87** (5) | | | 20.03±4.11 (10) | | 33.64± 17.42 (10) |
| Ta | **0.220±0.089** (7) | **0.790±0.166** (7) | | | 0.112±0.073 (4) | 0.397±0.051 (2) | | | **0.179±0.056** (4) | **0.826**(1) | | | 0.496±0.219 (10) | | 0.644±0.211 (7) |
| Tb | 0.475±0.017 (11) | 0.587±0.071 (11) | | | 0.559±0.017 (4) | 0.689±0.193 (4) | | | **0.451±0.031** (5) | **0.680±0.073** (5) | | | 0.885±0.193 (10) | | 0.590±0.093 (10) |
| Te | 1.17±0.173 (11) | 6.82±1.43 (10) | | | **0.611±0.198** (4) | **12.87±4.03** (4) | | | **1.45±0.276** (5) | **4.83±0.588** (5) | | | **1.36±0.316** (10) | | **3.97±1.121** (10) |
| Ti | 0.083±0.020 (6) | 0.135±0.037 (9) | | | 0.201±0.182 (3) | 0.752±0.336 (4) | | | 0.048±0.009 (2) | 0.330±0.109 (5) | | | 0.118±0.027 (9) | | 0.934±0.764 (10) |
| Tl | **0.551±0.162** (9) | **2.44±0.596** (7) | | | 0.916±0.309 (4) | 1.54±0.774 (4) | | | 0.639±0.214 (5) | 1.64±0.521 (4) | | | 0.892±0.141 (7) | | 0.850±0.300 (7) |
| Tm | **0.064±0.020** (7) | **1.172±0.171** (10) | | | BDL | 0.708±0.313 (4) | | | 0.062(1) | 1.078±0.403 (4) | | | 0.074±0.053 (3) | | 0.783±0.204 (9) |
| U | 0.501±0.144 (6) | BDL | | | 0.707±0.212 (2) | BDL | | | 0.368±0.024 (2) | BDL | | | 0.580±0.263 (3) | | BDL |
| V | 0.019±0.006 (2) | BDL | | | **0.030±0.012** (2) | **0.389** (1) | | | 0.033±0.012 (3) | BDL | | | 0.046±0.019 (6) | | 0.019 (1) |
| W | 0.135±0.059 (3) | 0.221±0.183 (2) | | | **0.298±0.016** (3) | **2.742±0.324** (2) | | | 0.323 (1) | 1.120±0.456 (3) | | | 0.871±0.437 (2) | | 0.560±0.215 (4) |
| Y | 0.013±0.001 (2) | 0.079±0.028 (5) | | | BDL | 0.156±0.074 (3) | | | 0.031 (1) | 0.085±0.014 (3) | | | BDL | | 0.041±0.029 (2) |
| Yb | BDL | 0.030±0.006 (6) | | | BDL | 0.042±0.018 (3) | | | BDL | 0.023±0.010 (2) | | | 0.029 (1) | | 0.022±0.006 (5) |
| Zn | 5.78±1.01 (11) | 5.83±1.05 (11) | | | 8.38±4.25 (4) | 9.97±5.28 (4) | | | 4.04±1.18 (5) | 5.50±1.00 (5) | | | 2.54±0.40 (10) | | 8.83±5.73 (10) |
| Zr | 0.086±0.026 (3) | 0.709±0.288 (6) | | | 0.932 (1) | 0.539±0.073 (4) | | | BDL | 0.491±0.108 (4) | | | 0.095±0.040 (2) | | 0.474±0.224 (6) |

Table S5. Average ± SE (sample size) ICP-OES-based elemental concentrations (ppm d.w.) measured in the background colour and pigment spot regions of the shells of non-embryonated eggs and post-hatched eggshells of wild and captive Capercaillie *Tetrao urogallus*. BDL – all samples below the detection limit. The averages in bold, differing significantly from each other (one-way ANOVA, *P* ≤ 0.05). *Note:* In the case of captive Capercaillies only post-hatched eggshells were examined.

| Element | Wild Capercaillie | |  | | |  | |  | Captive Capercaillie | |
| --- | --- | --- | --- | --- | --- | --- | --- | --- | --- | --- |
|  | Non-embryonated | | |  | Post-hatched | | |  | Post-hatched | |
|  | Background | Spot | |  | Background | | Spot |  | Background | Spot |
| Ag | 0.187±0.107 (3) | 0.671±0.330 (4) | | | **0.138±0.034** (2) | | **0.300±0.115** (4) | | 0.041±0.016 (4) | 0.280±0.189 (2) |
| Al | 13.68±1.319 (8) | 17.85±4.074 (8) | | | 21.79±1.822 (14) | | 37.93±5.330 (14) | | **24.18±4.519** (13) | **39.03±4.781** (13) |
| As | BDL | BDL | | | 1.503 (1) | | BDL | | BDL | BDL |
| B | 0.276±0.137 (3) | 0.357±0.242 (2) | | | **7.778±1.558** (14) | | **26.09±5.426** (14) | | **4.389±1.847** (10) | **25.42±9.013** (11) |
| Ba | 165.0±20.74 (8) | 152.9±32.11 (8) | | | 167.7±29.78 (14) | | 177.0±28.46 (14) | | 74.66±8.022 (13) | 80.11±4.001 (13) |
| Be | **0.127±0.011** (8) | **0.609±0.144** (8) | | | **0.044±0.004** (14) | | **0.200±0.022** (14) | | **0.070±0.013** (13) | **0.451±0.061** (13) |
| Cd | BDL | BDL | | | BDL | | BDL | | 10.38 (1) | 4.21 (1) |
| Cr | **0.890±0.131** (8) | **3.659±0.823** (8) | | | **0.365±0.023** (14) | | **1.233±0.113** (14) | | **0.714±0.172** (13) | **3.839±0.738** (13) |
| Dy | BDL | BDL | | | 0.089±0.018 (7) | | BDL | | 0.162±0.103 (2) | BDL |
| Fe | **20.81±3.245** (8) | **61.46±17.17** (8) | | | **19.82±1.300** (14) | | **39.07±4.229** (14) | | **25.48±3.663** (13) | **64.38±6.307** (13) |
| K | 114.6±50.92 (3) | BDL | | | **561.4±19.20** (14) | | **272.8±37.71** (8) | | 639.2±80.83 (13) | 485.2±90.98 (3) |
| Mn | 4.843±1.259 (8) | 6.604±1.919 (8) | | | 6.618±0.759 (14) | | 11.97±3.549 (14) | | 6.049±1.494 (13) | 9.248±2.560 (13) |
| Mo | **1.461±0.268** (7) | **7.044±1.695** (8) | | | **0.631±0.124** (13) | | **1.633±0.373** (12) | | 1.441±0.653 (12) | 9.269±4.221 (12) |
| Na | 2610.2±75.48 (8) | 3984.3±701.3 (8) | | | **1794.7±87.44** (14) | | **2601.6±154.4** (14) | | **2371.9±116.8** (13) | **4057.9±211.9** (13) |
| Ni | **7.267±1.324** (8) | **29.91±7.626** (8) | | | **3.256±0.321** (14) | | **10.16±1.226** (14) | | **5.119±1.465** (13) | **17.84±2.957** (13) |
| Pb | 4.061±0.953 (4) | 5.375 (1) | | | 2.320±0.590 (7) | | 4.200±2.081 (5) | | **2.078±0.925** (4) | **13.99±3.282** (3) |
| Re | 3.844±1.259 (6) | 7.314±2.230 (3) | | | **1.594±0.259** (11) | | **4.149±0.970** (13) | | 2.595±0.638 (10) | 3.796±0.680 (8) |
| Ru | **4.521±0.449** (8) | **22.656±6.889** (8) | | | **1.313±0.166** (14) | | **5.206±0.536** (14) | | **1.885±0.314** (13) | **14.58±1.955** (13) |
| Sb | BDL | BDL | | | BDL | | BDL | | 12.60 (1) | 177.7 (1) |
| Sm | **16.91±2.042** (8) | **113.5±29.52** (8) | | | **3.843±0.491** (14) | | **30.94±4.174** (14) | | **8.188±2.216** (13) | **79.77±12.41** (13) |
| Sn | BDL | BDL | | | 22.59±2.515 (12) | | 11.03 (1) | | 23.03±2.594 (9) | BDL |
| Ta | BDL | BDL | | | BDL | | BDL | | 22.38 (1) | 155.34 (1) |
| Tb | 1.191±0.120 (8) | 2.322±0.557 (8) | | | **0.816±0.056** (14) | | **1.440±0.088** (14) | | **0.901±0.119** (13) | **1.993±0.230** (13) |
| Th | BDL | BDL | | | 0.752±0.266 (7) | | BDL | | 0.929±0.349 (5) | BDL |
| W | **0.693±0.127** (3) | **2.066** (1) | | | 0.585 (1) | | 3.523±2.797 (2) | | 1.786±0.653 (6) | 1.663 (1) |
| Zn | 11.92±0.951 (8) | 26.21±5.156 (8) | | | **3.941±0.782** (14) | | **8.632±1.294** (14) | | **10.17±1.617** (13) | **23.06±3.454** (13) |
| Zr | 1.478±0.782 (3) | 3.572±1.648 (3) | | | BDL | | 16.220 (1) | | BDL | 9.392±6.990 (2) |

Table S6. Results of *t*-tests for paired comparisons and the accompanying statistics of the ICP-OES-based elemental concentrations (ppm d.w.) measured in the background colour and pigment spot regions of the same shell from non-embryonated eggs and post-hatched eggshells of Black Grouse *Tetrao tetrix*. BDL – all samples below the detection limit. The averages differing significantly from each other (*P* ≤ 0.05) are highlighted in red font.

| Element/  region | **ALL EGGSHELLS** | | | |  |  |  |  |  |  | **NON-EMBRYONATED** | | | | |  |  |  |  |  | **POST-HATCHED** | | | |  |  |  |  |  |  |
| --- | --- | --- | --- | --- | --- | --- | --- | --- | --- | --- | --- | --- | --- | --- | --- | --- | --- | --- | --- | --- | --- | --- | --- | --- | --- | --- | --- | --- | --- | --- |
|  | Aver | ±SD | n | Differ. | ±SD | t | df | *P*-value | -95% CI | +95% CI | Aver | ±SD | n | Differ. | ±SD | t | df | P-value | -95% CI | +95% CI | Aver | ±SD | n | Differ. | ±SD | t | df | P-value | -95% CI | +95% CI |
| Al-back | 16.28 | 11.55 |  |  |  |  |  |  |  |  | 10.67 | 4.44 |  |  |  |  |  |  |  |  | 22.69 | 13.84 |  |  |  |  |  |  |  |  |
| Al-spot | 25.90 | 12.73 | 30 | -9.620 | 19.13 | -2.75 | 29 | 0.0100 | -16.76 | -2.48 | 23.30 | 6.86 | 16 | -12.63 | 6.20 | -8.15 | 15 | 0.0000 | -15.94 | -9.33 | 28.87 | 17.01 | 14 | -6.176 | 27.349 | -0.845 | 13 | 0.413 | -21.97 | 9.62 |
| As-back | 1.335 | 0.882 |  |  |  |  |  |  |  |  | 1.337 | 1.248 |  |  |  |  |  |  |  |  | 1.333 | BDL |  |  |  |  |  |  |  |  |
| As-spot | 4.746 | 3.418 | 3 | -3.411 | 3.310 | -1.78 | 2 | 0.2162 | -11.63 | 4.81 | 2.837 | 1.218 | 2 | -1.50 | 0.03 | -70.91 | 1 | 0.0090 | -1.77 | -1.23 | 8.565 | BDL | 1 | -7.233 | - | - | - | - | - | - |
| B-back | 0.429 | 0.393 |  |  |  |  |  |  |  |  | 0.522 | 0.468 |  |  |  |  |  |  |  |  | 0.308 | 0.252 |  |  |  |  |  |  |  |  |
| B-spot | 1.162 | 1.371 | 16 | -0.734 | 1.083 | -2.71 | 15 | 0.0161 | -1.31 | -0.16 | 1.324 | 1.225 | 9 | -0.80 | 0.87 | -2.76 | 8 | 0.0245 | -1.47 | -0.13 | 0.954 | 1.614 | 7 | -0.646 | 1.380 | -1.238 | 6 | 0.262 | -1.92 | 0.63 |
| Ba-back | 357.3 | 208.8 |  |  |  |  |  |  |  |  | 390.2 | 239.5 |  |  |  |  |  |  |  |  | 319.7 | 167.8 |  |  |  |  |  |  |  |  |
| Ba-spot | 329.4 | 221.2 | 30 | 27.85 | 139.4 | 1.09 | 29 | 0.2828 | -24.20 | 79.89 | 407.7 | 240.8 | 16 | -17.49 | 36.02 | -1.94 | 15 | 0.071 | -36.69 | 1.70 | 240.0 | 161.6 | 14 | 79.7 | 190.8 | 1.562 | 13 | 0.142 | -30.52 | 189.9 |
| Cd-back | 0.029 | 0.013 |  |  |  |  |  |  |  |  | 0.028 | 0.015 |  |  |  |  |  |  |  |  | 0.030 | 0.015 |  |  |  |  |  |  |  |  |
| Cd-spot | 0.091 | 0.045 | 5 | -0.061 | 0.050 | -2.77 | 4 | 0.0505 | -0.12 | 0.00 | 0.064 | 0.041 | 2 | -0.04 | 0.03 | -1.99 | 1 | 0.297 | -0.27 | 0.20 | 0.108 | 0.045 | 3 | -0.078 | 0.059 | -2.278 | 2 | 0.150 | -0.23 | 0.07 |
| Ce-back | 0.632 | 0.498 |  |  |  |  |  |  |  |  | 0.499 | 0.248 |  |  |  |  |  |  |  |  | 0.798 | 0.674 |  |  |  |  |  |  |  |  |
| Ce-spot | 1.842 | 1.287 | 27 | -1.210 | 1.420 | -4.43 | 26 | 0.0002 | -1.77 | -0.65 | 2.170 | 1.186 | 15 | -1.67 | 1.21 | -5.34 | 14 | 0.0001 | -2.34 | -1.00 | 1.432 | 1.340 | 12 | -0.635 | 1.501 | -1.465 | 11 | 0.171 | -1.59 | 0.32 |
| Co-back | 0.151 | 0.114 |  |  |  |  |  |  |  |  | 0.130 | 0.109 |  |  |  |  |  |  |  |  | 0.181 | 0.119 |  |  |  |  |  |  |  |  |
| Co-spot | 0.561 | 0.526 | 26 | -0.410 | 0.550 | -3.80 | 25 | 0.0008 | -0.63 | -0.19 | 0.731 | 0.562 | 15 | -0.60 | 0.58 | -4.02 | 14 | 0.0013 | -0.92 | -0.28 | 0.330 | 0.385 | 11 | -0.149 | 0.396 | -1.248 | 10 | 0.240 | -0.41 | 0.12 |
| Cr-back | 0.181 | 0.155 |  |  |  |  |  |  |  |  | 0.097 | 0.035 |  |  |  |  |  |  |  |  | 0.278 | 0.183 |  |  |  |  |  |  |  |  |
| Cr-spot | 0.364 | 0.298 | 30 | -0.183 | 0.305 | -3.28 | 29 | 0.0027 | -0.30 | -0.07 | 0.347 | 0.204 | 16 | -0.25 | 0.21 | -4.84 | 15 | 0.0002 | -0.36 | -0.14 | 0.383 | 0.386 | 14 | -0.105 | 0.382 | -1.026 | 13 | 0.323 | -0.33 | 0.12 |
| Cu-back | 1.579 | 0.953 |  |  |  |  |  |  |  |  | 1.251 | 0.451 |  |  |  |  |  |  |  |  | 1.955 | 1.228 |  |  |  |  |  |  |  |  |
| Cu-spot | 3.454 | 4.102 | 30 | -1.875 | 3.962 | -2.59 | 29 | 0.0148 | -3.35 | -0.40 | 2.291 | 1.270 | 16 | -1.04 | 1.09 | -3.83 | 15 | 0.0016 | -1.62 | -0.46 | 4.783 | 5.667 | 14 | -2.828 | 5.641 | -1.876 | 13 | 0.083 | -6.09 | 0.43 |
| Fe-back | 24.21 | 19.20 |  |  |  |  |  |  |  |  | 19.63 | 16.31 |  |  |  |  |  |  |  |  | 29.44 | 21.45 |  |  |  |  |  |  |  |  |
| Fe-spot | 52.61 | 76.80 | 30 | -28.40 | 73.71 | -2.11 | 29 | 0.0436 | -55.92 | -0.88 | 36.64 | 36.60 | 16 | -17.01 | 28.68 | -2.37 | 15 | 0.0315 | -32.30 | -1.73 | 70.86 | 104.60 | 14 | -41.42 | 104.1 | -1.489 | 13 | 0.160 | -101.50 | 18.66 |
| Gd-back | 0.090 | 0.030 |  |  |  |  |  |  |  |  | 0.085 | 0.037 |  |  |  |  |  |  |  |  | 0.096 | 0.025 |  |  |  |  |  |  |  |  |
| Gd-spot | 0.060 | 0.050 | 7 | 0.030 | 0.074 | 1.09 | 6 | 0.3193 | -0.04 | 0.10 | 0.057 | 0.024 | 4 | 0.03 | 0.06 | 0.95 | 3 | 0.413 | -0.07 | 0.12 | 0.064 | 0.081 | 3 | 0.033 | 0.105 | 0.543 | 2 | 0.641 | -0.23 | 0.29 |
| Ge-back | 0.572 | 0.386 |  |  |  |  |  |  |  |  | 0.461 | - |  |  |  |  |  |  |  |  | 0.594 | 0.427 |  |  |  |  |  |  |  |  |
| Ge-spot | 0.618 | 0.911 | 6 | -0.046 | 1.085 | -0.10 | 5 | 0.9213 | -1.18 | 1.09 | 2.453 | - | 1 | -1.99 | - | - | - | - | - | - | 0.251 | 0.162 | 5 | 0.343 | 0.579 | 1.326 | 4 | 0.256 | -0.38 | 1.06 |
| Hf-back | 0.452 | 0.392 |  |  |  |  |  |  |  |  | 0.356 | 0.169 |  |  |  |  |  |  |  |  | 0.556 | 0.527 |  |  |  |  |  |  |  |  |
| Hf-spot | 1.517 | 1.134 | 29 | -1.065 | 1.176 | -4.88 | 28 | 0.0000 | -1.51 | -0.62 | 1.298 | 0.815 | 15 | -0.94 | 0.89 | -4.11 | 14 | 0.0011 | -1.43 | -0.45 | 1.753 | 1.393 | 14 | -1.197 | 1.446 | -3.097 | 13 | 0.0085 | -2.03 | -0.36 |
| In-back | 0.537 | 0.547 |  |  |  |  |  |  |  |  | 0.667 | 0.855 |  |  |  |  |  |  |  |  | 0.407 | 0.314 |  |  |  |  |  |  |  |  |
| In-spot | 0.873 | 0.502 | 4 | -0.336 | 0.866 | -0.78 | 3 | 0.4945 | -1.71 | 1.04 | 0.632 | 0.420 | 2 | 0.03 | 1.28 | 0.04 | 1 | 0.976 | -11.43 | 11.50 | 1.113 | 0.590 | 2 | -0.706 | 0.276 | -3.621 | 1 | 0.172 | -3.18 | 1.77 |
| K-back | 1204.0 | 739.2 |  |  |  |  |  |  |  |  | 918.2 | 131.0 |  |  |  |  |  |  |  |  | 1530.6 | 991.9 |  |  |  |  |  |  |  |  |
| K-spot | 1003.4 | 298.2 | 30 | 200.6 | 861.2 | 1.28 | 29 | 0.2121 | -120.96 | 522.2 | 1020.5 | 165.2 | 16 | -102.3 | 100.5 | -4.07 | 15 | 0.0010 | -155.87 | -48.80 | 983.8 | 407.6 | 14 | 546.830 | 1183.5 | 1.729 | 13 | 0.108 | -136.52 | 1230 |
| La-back | 0.280 | 0.250 |  |  |  |  |  |  |  |  | 0.188 | 0.108 |  |  |  |  |  |  |  |  | 0.385 | 0.322 |  |  |  |  |  |  |  |  |
| La-spot | 0.518 | 0.333 | 30 | -0.238 | 0.462 | -2.82 | 29 | 0.0085 | -0.41 | -0.07 | 0.483 | 0.316 | 16 | -0.30 | 0.36 | -3.28 | 15 | 0.0051 | -0.49 | -0.10 | 0.558 | 0.358 | 14 | -0.173 | 0.563 | -1.149 | 13 | 0.271 | -0.50 | 0.15 |
| Li-back | 0.469 | 1.194 |  |  |  |  |  |  |  |  | 0.113 | 0.031 |  |  |  |  |  |  |  |  | 0.876 | 1.721 |  |  |  |  |  |  |  |  |
| Li-spot | 0.254 | 0.381 | 15 | 0.215 | 0.841 | 0.99 | 14 | 0.3399 | -0.25 | 0.68 | 0.142 | 0.097 | 8 | -0.03 | 0.08 | -1.04 | 7 | 0.3317 | -0.10 | 0.04 | 0.382 | 0.541 | 7 | 0.494 | 1.214 | 1.076 | 6 | 0.323 | -0.63 | 1.62 |
| Lu-back | 0.080 | 0.070 |  |  |  |  |  |  |  |  | 0.063 | 0.021 |  |  |  |  |  |  |  |  | 0.098 | 0.097 |  |  |  |  |  |  |  |  |
| Lu-spot | 0.238 | 0.192 | 29 | -0.158 | 0.223 | -3.81 | 28 | 0.0007 | -0.24 | -0.07 | 0.228 | 0.180 | 15 | -0.16 | 0.19 | -3.39 | 14 | 0.0044 | -0.27 | -0.06 | 0.248 | 0.210 | 14 | -0.150 | 0.263 | -2.144 | 13 | 0.051 | -0.30 | 0.00 |
| Mg-back | 3099.0 | 1955.0 |  |  |  |  |  |  |  |  | 2449.5 | 551.4 |  |  |  |  |  |  |  |  | 3841.2 | 2657.7 |  |  |  |  |  |  |  |  |
| Mg-spot | 3401.3 | 954.5 | 30 | -302.4 | 2547.6 | -0.65 | 29 | 0.5208 | -1253.7 | 648.92 | 3715.7 | 622.9 | 16 | -1266.1 | 534.6 | -9.47 | 15 | 0.0000 | -1551.0 | -981.2 | 3042.1 | 1150.7 | 14 | 799.056 | 3420.4 | 0.874 | 13 | 0.398 | -1175.8 | 2774 |
| Mn-back | 10.43 | 9.36 |  |  |  |  |  |  |  |  | 11.40 | 11.42 |  |  |  |  |  |  |  |  | 9.325 | 6.514 |  |  |  |  |  |  |  |  |
| Mn-spot | 13.63 | 13.31 | 30 | -3.202 | 8.638 | -2.03 | 29 | 0.0516 | -6.43 | 0.02 | 17.75 | 15.96 | 16 | -6.35 | 7.69 | -3.30 | 15 | 0.0048 | -10.45 | -2.25 | 8.924 | 7.541 | 14 | 0.400 | 8.480 | 0.177 | 13 | 0.862 | -4.50 | 5.30 |
| Mo-back | 0.120 | 0.070 |  |  |  |  |  |  |  |  | BDL | - |  |  |  |  |  |  |  |  | 0.120 | 0.070 |  |  |  |  |  |  |  |  |
| Mo-spot | 0.261 | 0.039 | 4 | -0.141 | 0.085 | -3.30 | 3 | 0.0456 | -0.28 | -0.01 | BDL | - | - | - | - | - | - | - | - | - | 0.261 | 0.039 | 4 | -0.141 | 0.085 | -3.303 | 3 | 0.046 | -0.28 | -0.01 |
| Na-back | 1268.3 | 611.9 |  |  |  |  |  |  |  |  | 1110.3 | 111.4 |  |  |  |  |  |  |  |  | 1448.8 | 869.0 |  |  |  |  |  |  |  |  |
| Na-spot | 1072.8 | 315.4 | 30 | 195.4 | 785.5 | 1.36 | 29 | 0.1835 | -97.91 | 488.7 | 1179.4 | 134.0 | 16 | -69.09 | 140.0 | -1.97 | 15 | 0.067 | -143.67 | 5.50 | 951.1 | 413.8 | 14 | 497.706 | 1081.4 | 1.722 | 13 | 0.109 | -126.7 | 1122 |
| Nd-back | 1.248 | 0.713 |  |  |  |  |  |  |  |  | 1.090 | 0.492 |  |  |  |  |  |  |  |  | 1.428 | 0.890 |  |  |  |  |  |  |  |  |
| Nd-spot | 3.698 | 2.212 | 30 | -2.450 | 2.517 | -5.33 | 29 | 0.0000 | -3.39 | -1.51 | 4.352 | 2.419 | 16 | -3.26 | 2.66 | -4.90 | 15 | 0.0002 | -4.68 | -1.84 | 2.951 | 1.744 | 14 | -1.523 | 2.058 | -2.770 | 13 | 0.016 | -2.71 | -0.34 |
| Ni-back | 0.556 | 0.440 |  |  |  |  |  |  |  |  | 0.320 | 0.152 |  |  |  |  |  |  |  |  | 0.811 | 0.510 |  |  |  |  |  |  |  |  |
| Ni-spot | 1.060 | 1.332 | 25 | -0.504 | 1.361 | -1.85 | 24 | 0.0764 | -1.07 | 0.06 | 0.840 | 0.439 | 13 | -0.52 | 0.50 | -3.74 | 12 | 0.0028 | -0.82 | -0.22 | 1.298 | 1.882 | 12 | -0.487 | 1.942 | -0.869 | 11 | 0.403 | -1.72 | 0.75 |
| Pb-back | 2.237 | 1.635 |  |  |  |  |  |  |  |  | 1.696 | 0.600 |  |  |  |  |  |  |  |  | 2.855 | 2.186 |  |  |  |  |  |  |  |  |
| Pb-spot | 4.691 | 3.148 | 30 | -2.454 | 3.738 | -3.60 | 29 | 0.0012 | -3.85 | -1.06 | 4.313 | 2.281 | 16 | -2.62 | 2.15 | -4.87 | 15 | 0.0002 | -3.76 | -1.47 | 5.122 | 3.965 | 14 | -2.267 | 5.076 | -1.671 | 13 | 0.119 | -5.20 | 0.66 |
| Pr-back | 44.11 | 27.24 |  |  |  |  |  |  |  |  | 34.925 | 2.128 |  |  |  |  |  |  |  |  | 54.60 | 37.78 |  |  |  |  |  |  |  |  |
| Pr-spot | 34.13 | 8.69 | 30 | 9.977 | 31.83 | 1.72 | 29 | 0.0967 | -1.91 | 21.86 | 36.186 | 3.412 | 16 | -1.26 | 3.34 | -1.51 | 15 | 0.152 | -3.04 | 0.52 | 31.78 | 12.00 | 14 | 22.820 | 43.758 | 1.951 | 13 | 0.073 | -2.44 | 48.09 |
| Re-back | 0.362 | 0.182 |  |  |  |  |  |  |  |  | 0.332 | 0.239 |  |  |  |  |  |  |  |  | 0.384 | 0.145 |  |  |  |  |  |  |  |  |
| Re-spot | 1.182 | 1.248 | 12 | -0.819 | 1.233 | -2.30 | 11 | 0.0418 | -1.60 | -0.04 | 1.167 | 1.168 | 5 | -0.84 | 1.12 | -1.66 | 4 | 0.172 | -2.23 | 0.56 | 1.192 | 1.394 | 7 | -0.808 | 1.394 | -1.533 | 6 | 0.176 | -2.10 | 0.48 |
| Rh-back | 0.335 | 0.201 |  |  |  |  |  |  |  |  | 0.255 | 0.139 |  |  |  |  |  |  |  |  | 0.415 | 0.225 |  |  |  |  |  |  |  |  |
| Rh-spot | 0.445 | 0.357 | 28 | -0.110 | 0.439 | -1.32 | 27 | 0.1968 | -0.28 | 0.06 | 0.539 | 0.375 | 14 | -0.28 | 0.42 | -2.52 | 13 | 0.0257 | -0.53 | -0.04 | 0.350 | 0.325 | 14 | 0.065 | 0.395 | 0.613 | 13 | 0.550 | -0.16 | 0.29 |
| Ru-back | 0.228 | 0.263 |  |  |  |  |  |  |  |  | 0.154 | 0.105 |  |  |  |  |  |  |  |  | 0.316 | 0.364 |  |  |  |  |  |  |  |  |
| Ru-spot | 0.902 | 0.853 | 22 | -0.674 | 0.951 | -3.32 | 21 | 0.0032 | -1.10 | -0.25 | 1.075 | 0.984 | 12 | -0.92 | 1.03 | -3.10 | 11 | 0.0101 | -1.57 | -0.27 | 0.694 | 0.654 | 10 | -0.377 | 0.800 | -1.491 | 9 | 0.170 | -0.95 | 0.20 |
| Sb-back | 17.99 | 13.14 |  |  |  |  |  |  |  |  | 14.62 | 9.95 |  |  |  |  |  |  |  |  | 24.75 | 17.62 |  |  |  |  |  |  |  |  |
| Sb-spot | 24.98 | 23.73 | 12 | -6.983 | 20.92 | -1.16 | 11 | 0.2721 | -20.28 | 6.31 | 24.58 | 19.70 | 8 | -9.96 | 22.54 | -1.25 | 7 | 0.251 | -28.80 | 8.88 | 25.78 | 34.03 | 4 | -1.025 | 18.678 | -0.110 | 3 | 0.920 | -30.75 | 28.70 |
| Sc-back | 0.125 | 0.077 |  |  |  |  |  |  |  |  | 0.099 | 0.036 |  |  |  |  |  |  |  |  | 0.155 | 0.100 |  |  |  |  |  |  |  |  |
| Sc-spot | 0.111 | 0.035 | 30 | 0.014 | 0.093 | 0.82 | 29 | 0.4191 | -0.02 | 0.05 | 0.114 | 0.029 | 16 | -0.02 | 0.04 | -1.79 | 15 | 0.094 | -0.03 | 0.00 | 0.107 | 0.042 | 14 | 0.048 | 0.124 | 1.435 | 13 | 0.175 | -0.02 | 0.12 |
| Se-back | 1.435 | 1.227 |  |  |  |  |  |  |  |  | 0.899 | 0.577 |  |  |  |  |  |  |  |  | 2.090 | 1.509 |  |  |  |  |  |  |  |  |
| Se-spot | 2.961 | 2.933 | 20 | -1.526 | 3.633 | -1.88 | 19 | 0.0758 | -3.23 | 0.17 | 3.361 | 2.842 | 11 | -2.46 | 3.07 | -2.66 | 10 | 0.0240 | -4.53 | -0.40 | 2.471 | 3.138 | 9 | -0.381 | 4.107 | -0.278 | 8 | 0.788 | -3.54 | 2.78 |
| Si-back | 12.79 | 9.53 |  |  |  |  |  |  |  |  | 7.888 | 3.208 |  |  |  |  |  |  |  |  | 18.393 | 11.284 |  |  |  |  |  |  |  |  |
| Si-spot | 29.01 | 34.31 | 30 | -16.2 | 36.39 | -2.44 | 29 | 0.0210 | -29.81 | -2.63 | 21.686 | 9.214 | 16 | -13.80 | 8.03 | -6.88 | 15 | 0.0000 | -18.08 | -9.52 | 37.380 | 48.858 | 14 | -18.988 | 53.511 | -1.328 | 13 | 0.207 | -49.88 | 11.91 |
| Ta-back | 0.372 | 0.605 |  |  |  |  |  |  |  |  | 0.213 | 0.255 |  |  |  |  |  |  |  |  | 0.478 | 0.753 |  |  |  |  |  |  |  |  |
| Ta-spot | 0.649 | 0.468 | 15 | -0.277 | 0.861 | -1.25 | 14 | 0.2322 | -0.75 | 0.20 | 0.739 | 0.451 | 6 | -0.53 | 0.57 | -2.25 | 5 | 0.0738 | -1.13 | 0.07 | 0.589 | 0.496 | 9 | -0.111 | 1.007 | -0.331 | 8 | 0.749 | -0.89 | 0.66 |
| Tb-back | 0.619 | 0.393 |  |  |  |  |  |  |  |  | 0.467 | 0.061 |  |  |  |  |  |  |  |  | 0.792 | 0.530 |  |  |  |  |  |  |  |  |
| Tb-spot | 0.617 | 0.259 | 30 | 0.002 | 0.502 | 0.02 | 29 | 0.9851 | -0.19 | 0.19 | 0.616 | 0.214 | 16 | -0.15 | 0.20 | -3.01 | 15 | 0.0087 | -0.25 | -0.04 | 0.618 | 0.311 | 14 | 0.174 | 0.677 | 0.960 | 13 | 0.354 | -0.22 | 0.56 |
| Te-back | 1.201 | 0.762 |  |  |  |  |  |  |  |  | 1.250 | 0.603 |  |  |  |  |  |  |  |  | 1.149 | 0.924 |  |  |  |  |  |  |  |  |
| Te-spot | 6.328 | 5.137 | 29 | -5.127 | 5.293 | -5.22 | 28 | 0.0000 | -7.14 | -3.11 | 6.154 | 3.817 | 15 | -4.90 | 4.08 | -4.66 | 14 | 0.0004 | -7.16 | -2.65 | 6.515 | 6.409 | 14 | -5.366 | 6.505 | -3.087 | 13 | 0.0087 | -9.12 | -1.61 |
| Ti-back | 0.113 | 0.126 |  |  |  |  |  |  |  |  | 0.074 | 0.046 |  |  |  |  |  |  |  |  | 0.139 | 0.156 |  |  |  |  |  |  |  |  |
| Ti-spot | 0.611 | 1.701 | 20 | -0.498 | 1.727 | -1.29 | 19 | 0.213 | -1.31 | 0.31 | 0.221 | 0.218 | 8 | -0.15 | 0.24 | -1.76 | 7 | 0.122 | -0.34 | 0.05 | 0.871 | 2.187 | 12 | -0.732 | 2.229 | -1.138 | 11 | 0.279 | -2.15 | 0.68 |
| Tl-back | 0.752 | 0.502 |  |  |  |  |  |  |  |  | 0.615 | 0.512 |  |  |  |  |  |  |  |  | 0.904 | 0.472 |  |  |  |  |  |  |  |  |
| Tl-spot | 1.548 | 1.175 | 19 | -0.796 | 1.300 | -2.67 | 18 | 0.0156 | -1.42 | -0.17 | 1.867 | 1.105 | 10 | -1.25 | 1.12 | -3.54 | 9 | 0.0064 | -2.05 | -0.45 | 1.193 | 1.209 | 9 | -0.289 | 1.357 | -0.639 | 8 | 0.540 | -1.33 | 0.75 |
| Tm-back | 0.057 | 0.051 |  |  |  |  |  |  |  |  | 0.069 | 0.055 |  |  |  |  |  |  |  |  | 0.021 | 0.004 |  |  |  |  |  |  |  |  |
| Tm-spot | 1.225 | 0.599 | 8 | -1.168 | 0.621 | -5.32 | 7 | 0.0011 | -1.69 | -0.65 | 1.085 | 0.639 | 6 | -1.02 | 0.66 | -3.80 | 5 | 0.0127 | -1.70 | -0.33 | 1.645 | 0.025 | 2 | -1.624 | 0.029 | -78.3 | 1 | 0.0081 | -1.89 | -1.36 |
| V-back | 0.021 | 0.005 |  |  |  |  |  |  |  |  | BDL | - |  |  |  |  |  |  |  |  | 0.021 | 0.005 |  |  |  |  |  |  |  |  |
| V-spot | 0.204 | 0.262 | 2 | -0.183 | 0.267 | -0.97 | 1 | 0.509 | -2.58 | 2.21 | BDL | - | - | - | - | - | - | - | - | - | 0.204 | 0.262 | 2 | -0.183 | 0.267 | -0.972 | 1 | 0.509 | -2.58 | 2.21 |
| W-back | 0.537 | 0.434 |  |  |  |  |  |  |  |  | 0.323 | - |  |  |  |  |  |  |  |  | 0.590 | 0.482 |  |  |  |  |  |  |  |  |
| W-spot | 1.766 | 1.013 | 5 | -1.229 | 1.372 | -2.00 | 4 | 0.116 | -2.93 | 0.47 | 1.734 | - | - | - | - | - | - | - | - | - | 1.775 | 1.170 | 4 | -1.184 | 1.579 | -1.499 | 3 | 0.231 | -3.70 | 1.33 |
| Y-back | 0.022 | 0.012 |  |  |  |  |  |  |  |  | 0.022 | 0.012 |  |  |  |  |  |  |  |  | BDL | BDL |  |  |  |  |  |  |  |  |
| Y-spot | 0.081 | 0.013 | 2 | -0.059 | 0.025 | -3.32 | 1 | 0.186 | -0.29 | 0.17 | 0.081 | 0.013 | 2 | -0.06 | 0.03 | -3.32 | 1 | 0.186 | -0.29 | 0.17 | BDL | BDL | - | - | - | - | - | - | - | - |
| Zn-back | 4.757 | 4.092 |  |  |  |  |  |  |  |  | 5.235 | 3.156 |  |  |  |  |  |  |  |  | 4.212 | 5.026 |  |  |  |  |  |  |  |  |
| Zn-spot | 7.327 | 11.02 | 30 | -2.569 | 10.85 | -1.30 | 29 | 0.205 | -6.62 | 1.48 | 5.726 | 3.064 | 16 | -0.49 | 2.42 | -0.81 | 15 | 0.429 | -1.78 | 0.80 | 9.16 | 15.92 | 14 | -4.944 | 15.634 | -1.183 | 13 | 0.258 | -13.97 | 4.08 |
| Zr-back | 0.249 | 0.384 |  |  |  |  |  |  |  |  | 0.086 | 0.045 |  |  |  |  |  |  |  |  | 0.494 | 0.620 |  |  |  |  |  |  |  |  |
| Zr-spot | 0.332 | 0.172 | 5 | -0.083 | 0.385 | -0.48 | 4 | 0.656 | -0.56 | 0.40 | 0.369 | 0.211 | 3 | -0.28 | 0.17 | -2.80 | 2 | 0.107 | -0.72 | 0.15 | 0.277 | 0.139 | 2 | 0.217 | 0.482 | 0.637 | 1 | 0.639 | -4.11 | 4.54 |

Table S7. Results of *t*-tests for paired comparisons and the accompanying statistics of the ICP-OES-based elemental concentrations (ppm d.w.) measured in the background colour and pigment spot regions of the same shell from non-embryonated eggs and post-hatched eggshells of Capercaillie *Tetrao urogallus*. BDL – all samples below the detection limit. The averages differing significantly from each other (*P* ≤ 0.05) are highlighted in red font.

| Element/  region | **ALL EGGSHELLS** | | | |  |  |  |  |  |  | **NON-EMBRYONATED** | | | | |  |  |  |  |  | **POST-HATCHED** | | | |  |  |  |  |  |  |
| --- | --- | --- | --- | --- | --- | --- | --- | --- | --- | --- | --- | --- | --- | --- | --- | --- | --- | --- | --- | --- | --- | --- | --- | --- | --- | --- | --- | --- | --- | --- |
|  | Aver | ±SD | n | Differ. | ±SD | t | df | P-value | -95% CI | +95% CI | Aver | ±SD | n | Differ. | ±SD | t | df | P-value | -95% CI | +95% CI | Aver | ±SD | n | Differ. | ±SD | t | df | P-value | -95% CI | +95% CI |
| Ag-back | 0.048 | 0.048 |  |  |  |  |  |  |  |  | BDL | - |  |  |  |  |  |  |  |  | 0.062 | 0.059 |  |  |  |  |  |  |  |  |
| Ag-spot | 0.402 | 0.089 | 3 | -0.354 | 0.087 | -7.08 | 2 | 0.0194 | -0.569 | -0.139 | BDL | - | - | - | - | - | - | - | - | - | 0.453 | 0.023 | 2 | -0.391 | 0.082 | -6.72 | 1 | 0.0941 | -1.131 | 0.349 |
| Al-back | 20.82 | 11.45 |  |  |  |  |  |  |  |  | 13.68 | 3.731 |  |  |  |  |  |  |  |  | 22.94 | 12.14 |  |  |  |  |  |  |  |  |
| Al-spot | 33.75 | 19.02 | 35 | -12.93 | 20.20 | -3.79 | 34 | 0.0006 | -19.87 | -5.99 | 17.85 | 11.52 | 8 | -4.173 | 12.107 | -0.975 | 7 | 0.3621 | -14.30 | 5.95 | 38.46 | 18.34 | 27 | -15.521 | 21.53 | -3.75 | 26 | 0.0009 | -24.037 | -7.005 |
| B-back | 5.904 | 5.943 |  |  |  |  |  |  |  |  | 0.354 | 0.275 |  |  |  |  |  |  |  |  | 6.366 | 5.957 |  |  |  |  |  |  |  |  |
| B-spot | 24.71 | 24.53 | 26 | -18.81 | 20.13 | -4.76 | 25 | 0.0001 | -26.94 | -10.68 | 0.357 | 0.342 | 2 | -0.002 | 0.067 | -0.047 | 1 | 0.9704 | -0.603 | 0.599 | 26.74 | 24.46 | 24 | -20.374 | 20.18 | -4.95 | 23 | 0.0001 | -28.894 | -11.854 |
| Ba-back | 132.5 | 88.26 |  |  |  |  |  |  |  |  | 165.0 | 58.67 |  |  |  |  |  |  |  |  | 122.9 | 94.02 |  |  |  |  |  |  |  |  |
| Ba-spot | 135.5 | 89.79 | 35 | -2.994 | 33.49 | -0.53 | 34 | 0.6004 | -14.50 | 8.51 | 152.9 | 90.83 | 8 | 12.047 | 60.948 | 0.559 | 7 | 0.5935 | -38.91 | 63.00 | 130.4 | 90.56 | 27 | -7.450 | 19.41 | -1.99 | 26 | 0.0566 | -15.127 | 0.226 |
| Be-back | 0.073 | 0.046 |  |  |  |  |  |  |  |  | 0.127 | 0.033 |  |  |  |  |  |  |  |  | 0.057 | 0.036 |  |  |  |  |  |  |  |  |
| Be-spot | 0.387 | 0.285 | 35 | -0.314 | 0.263 | -7.06 | 34 | 0.0000 | -0.404 | -0.224 | 0.609 | 0.407 | 8 | -0.482 | 0.408 | -3.341 | 7 | 0.0124 | -0.824 | -0.141 | 0.321 | 0.205 | 27 | -0.264 | 0.185 | -7.41 | 26 | 0.0000 | -0.337 | -0.191 |
| Cr-back | 0.615 | 0.462 |  |  |  |  |  |  |  |  | 0.890 | 0.371 |  |  |  |  |  |  |  |  | 0.533 | 0.461 |  |  |  |  |  |  |  |  |
| Cr-spot | 2.755 | 2.297 | 35 | -2.141 | 2.162 | -5.86 | 34 | 0.0000 | -2.883 | -1.398 | 3.659 | 2.329 | 8 | -2.768 | 2.378 | -3.292 | 7 | 0.0133 | -4.756 | -0.780 | 2.488 | 2.262 | 27 | -1.955 | 2.105 | -4.82 | 26 | 0.0001 | -2.788 | -1.122 |
| Fe-back | 22.15 | 9.739 |  |  |  |  |  |  |  |  | 20.81 | 9.179 |  |  |  |  |  |  |  |  | 22.55 | 10.03 |  |  |  |  |  |  |  |  |
| Fe-spot | 53.59 | 30.159 | 35 | -31.44 | 29.44 | -6.32 | 34 | 0.0000 | -41.55 | -21.33 | 61.46 | 48.56 | 8 | -40.66 | 51.06 | -2.252 | 7 | 0.0590 | -83.34 | 2.03 | 51.26 | 23.02 | 27 | -28.71 | 19.94 | -7.48 | 26 | 0.0000 | -36.598 | -20.819 |
| K-back | 693.3 | 258.6 |  |  |  |  |  |  |  |  | BDL | - |  |  |  |  |  |  |  |  | 693.3 | 258.6 |  |  |  |  |  |  |  |  |
| K-spot | 330.7 | 150.9 | 11 | 362.6 | 180.3 | 6.67 | 10 | 0.0001 | 241.5 | 483.76 | BDL | - | - | - | - | - | - | - | - | - | 330.7 | 150.9 | 11 | 362.6 | 180.3 | 6.67 | 10 | 0.0001 | 241.449 | 483.756 |
| Mn-back | 6.001 | 4.050 |  |  |  |  |  |  |  |  | 4.843 | 3.560 |  |  |  |  |  |  |  |  | 6.344 | 4.184 |  |  |  |  |  |  |  |  |
| Mn-spot | 9.730 | 10.39 | 35 | -3.729 | 8.472 | -2.60 | 34 | 0.0136 | -6.640 | -0.819 | 6.604 | 5.427 | 8 | -1.760 | 2.881 | -1.728 | 7 | 0.1276 | -4.17 | 0.65 | 10.66 | 11.38 | 27 | -4.313 | 9.491 | -2.36 | 26 | 0.0260 | -8.067 | -0.558 |
| Mo-back | 1.147 | 1.531 |  |  |  |  |  |  |  |  | 1.461 | 0.710 |  |  |  |  |  |  |  |  | 1.047 | 1.714 |  |  |  |  |  |  |  |  |
| Mo-spot | 6.290 | 10.06 | 29 | -5.143 | 10.00 | -2.77 | 28 | 0.0099 | -8.947 | -1.338 | 7.595 | 4.896 | 7 | -6.133 | 4.847 | -3.348 | 6 | 0.0155 | -10.62 | -1.65 | 5.875 | 11.28 | 22 | -4.827 | 11.24 | -2.02 | 21 | 0.0569 | -9.809 | 0.155 |
| Na-back | 2195.5 | 481.1 |  |  |  |  |  |  |  |  | 2610.2 | 213.5 |  |  |  |  |  |  |  |  | 2072.6 | 471.0 |  |  |  |  |  |  |  |  |
| Na-spot | 3458.6 | 1283.9 | 35 | -1263.1 | 1120.0 | -6.67 | 34 | 0.0000 | -1647.8 | -878.3 | 3984.3 | 1983.5 | 8 | -1374.1 | 2066.9 | -1.880 | 7 | 0.1021 | -3102.1 | 353.9 | 3302.8 | 993.0 | 27 | -1230.2 | 696.6 | -9.18 | 26 | 0.0000 | -1505.7 | -954.6 |
| Ni-back | 4.865 | 3.966 |  |  |  |  |  |  |  |  | 7.267 | 3.745 |  |  |  |  |  |  |  |  | 4.153 | 3.807 |  |  |  |  |  |  |  |  |
| Ni-spot | 17.527 | 14.23 | 35 | -12.66 | 12.77 | -5.87 | 34 | 0.0000 | -17.05 | -8.276 | 29.91 | 21.57 | 8 | -22.647 | 21.379 | -2.996 | 7 | 0.0200 | -40.52 | -4.77 | 13.856 | 8.850 | 27 | -9.703 | 7.097 | -7.10 | 26 | 0.0000 | -12.511 | -6.896 |
| Pb-back | 3.565 | 1.318 |  |  |  |  |  |  |  |  | BDL | - |  |  |  |  |  |  |  |  | 3.565 | 1.318 |  |  |  |  |  |  |  |  |
| Pb-spot | 11.492 | 7.651 | 3 | -7.927 | 6.335 | -2.17 | 2 | 0.1625 | -23.66 | 7.81 | BDL | - | - | - | - | - | - | - | - | - | 11.492 | 7.651 | 3 | -7.927 | 6.335 | -2.17 | 2 | 0.1625 | -23.664 | 7.810 |
| Re-back | 1.992 | 1.439 |  |  |  |  |  |  |  |  | 2.699 | 2.058 |  |  |  |  |  |  |  |  | 1.903 | 1.410 |  |  |  |  |  |  |  |  |
| Re-spot | 4.251 | 3.047 | 18 | -2.259 | 3.578 | -2.68 | 17 | 0.0159 | -4.038 | -0.48 | 5.407 | 2.832 | 2 | -2.708 | 4.889 | -0.783 | 1 | 0.5770 | -46.63 | 41.22 | 4.106 | 3.129 | 16 | -2.203 | 3.590 | -2.45 | 15 | 0.0268 | -4.116 | -0.290 |
| Ru-back | 2.259 | 1.599 |  |  |  |  |  |  |  |  | 4.521 | 1.271 |  |  |  |  |  |  |  |  | 1.588 | 0.932 |  |  |  |  |  |  |  |  |
| Ru-spot | 12.68 | 12.04 | 35 | -10.42 | 11.70 | -5.27 | 34 | 0.0000 | -14.44 | -6.40 | 22.66 | 19.48 | 8 | -18.14 | 20.070 | -2.556 | 7 | 0.0378 | -34.91 | -1.36 | 9.721 | 6.909 | 27 | -8.133 | 6.848 | -6.17 | 26 | 0.0000 | -10.842 | -5.424 |
| Sm-back | 8.443 | 7.502 |  |  |  |  |  |  |  |  | 16.91 | 5.775 |  |  |  |  |  |  |  |  | 5.935 | 6.003 |  |  |  |  |  |  |  |  |
| Sm-spot | 67.95 | 57.80 | 35 | -59.51 | 54.14 | -6.50 | 34 | 0.0000 | -78.11 | -40.91 | 113.5 | 83.48 | 8 | -96.61 | 84.024 | -3.252 | 7 | 0.0140 | -166.9 | -26.36 | 54.45 | 40.79 | 27 | -48.517 | 37.20 | -6.78 | 26 | 0.0000 | -63.232 | -33.801 |
| Tb-back | 0.933 | 0.356 |  |  |  |  |  |  |  |  | 1.191 | 0.339 |  |  |  |  |  |  |  |  | 0.857 | 0.330 |  |  |  |  |  |  |  |  |
| Tb-spot | 1.847 | 0.962 | 35 | -0.914 | 0.991 | -5.46 | 34 | 0.0000 | -1.25 | -0.57 | 2.322 | 1.576 | 8 | -1.131 | 1.678 | -1.907 | 7 | 0.0982 | -2.53 | 0.27 | 1.706 | 0.672 | 27 | -0.850 | 0.712 | -6.20 | 26 | 0.0000 | -1.131 | -0.568 |
| W-back | 2.644 | 2.431 |  |  |  |  |  |  |  |  | BDL | - |  |  |  |  |  |  |  |  | 2.903 | 2.996 |  |  |  |  |  |  |  |  |
| W-spot | 1.865 | 0.285 | 2 | 0.780 | 2.716 | 0.41 | 1 | 0.7545 | -23.62 | 25.18 | BDL | - | - | - | - | - | - | - | - | - | 2.903 | 2.996 | 3 | 0.000 | 0.000 | 0.00 | 2 | 1.0000 | 0.000 | 0.000 |
| Zn-back | 8.079 | 5.381 |  |  |  |  |  |  |  |  | 11.92 | 2.690 |  |  |  |  |  |  |  |  | 6.942 | 5.481 |  |  |  |  |  |  |  |  |
| Zn-spot | 18.010 | 13.01 | 35 | -9.931 | 10.69 | -5.49 | 34 | 0.0000 | -13.60 | -6.26 | 26.21 | 14.582 | 8 | -14.30 | 16.889 | -2.394 | 7 | 0.0479 | -28.42 | -0.18 | 15.58 | 11.72 | 27 | -8.637 | 8.073 | -5.56 | 26 | 0.0000 | -11.830 | -5.443 |
| Zr-back | 2.020 | 1.380 |  |  |  |  |  |  |  |  | 2.020 | 1.380 |  |  |  |  |  |  |  |  | BDL | - |  |  |  |  |  |  |  |  |
| Zr-spot | 5.134 | 1.288 | 2 | -3.114 | 0.092 | -47.98 | 1 | 0.0133 | -3.94 | -2.29 | 5.134 | 1.288 | 2 | -3.114 | 0.092 | -47.983 | 1 | 0.0133 | -3.94 | -2.29 | BDL | - | - | - | - | - | - | - | - | - |
